# Polyphyletic origin, intracellular invasion, and meiotic genes in the putatively asexual agamococcidians (Apicomplexa *incertae sedis*)

**DOI:** 10.1038/s41598-020-72287-x

**Published:** 2020-09-28

**Authors:** Tatiana S. Miroliubova, Timur G. Simdyanov, Kirill V. Mikhailov, Vladimir V. Aleoshin, Jan Janouškovec, Polina A. Belova, Gita G. Paskerova

**Affiliations:** 1 Severtsov Institute of Ecology and Evolution, Russian Academy of Sciences, Leninsky pr. 33, Moscow, 119071 Russian Federation; 2 Department of Invertebrate Zoology, Faculty of Biology, Saint Petersburg State University, Universitetskaya emb. 7/9, 199034 Saint Petersburg, Russian Federation; 3 Faculty of Biology, u1. Leninskiye Gory, Lomonosov Moscow State University, 1c12, 119991 Moscow, Russian Federation; 4 Belozersky Institute for Physico-Chemical Biology, Lomonosov Moscow State University, u1. Leninskiye Gory, 1c40, 119992 Moscow, Russian Federation; 5 Kharkevich Institute for Information Transmission Problems, Russian Academy of Sciences, Bolshoy Karetny per. 19c1, 127051 Moscow, Russian Federation; 6 Department of Pharmacy, University of Oslo, Sem Sælands vei 2C, 0371 Oslo, Norway

**Keywords:** Phylogenetics, Taxonomy, Parasite development, Parasite evolution, Parasite genomics

## Abstract

Agamococcidians are enigmatic and poorly studied parasites of marine invertebrates with unexplored diversity and unclear relationships to other sporozoans such as the human pathogens *Plasmodium* and *Toxoplasma*. It is believed that agamococcidians are not capable of sexual reproduction, which is essential for life cycle completion in all well studied parasitic apicomplexans. Here, we describe three new species of agamococcidians belonging to the genus *Rhytidocystis*. We examined their cell morphology and ultrastructure, resolved their phylogenetic position by using near-complete rRNA operon sequences, and searched for genes associated with meiosis and oocyst wall formation in two rhytidocystid transcriptomes. Phylogenetic analyses consistently recovered rhytidocystids as basal coccidiomorphs and away from the corallicolids, demonstrating that the order Agamococcidiorida Levine, 1979 is polyphyletic. Light and transmission electron microscopy revealed that the development of rhytidocystids begins inside the gut epithelial cells, a characteristic which links them specifically with other coccidiomorphs to the exclusion of gregarines and suggests that intracellular invasion evolved early in the coccidiomorphs. We propose a new superorder Eococcidia for early coccidiomorphs. Transcriptomic analysis demonstrated that both the meiotic machinery and oocyst wall proteins are preserved in rhytidocystids. The conservation of meiotic genes and ultrastructural similarity of rhytidocystid trophozoites to macrogamonts of true coccidians point to an undescribed, cryptic sexual process in the group.

## Introduction

The order Agamococcidiorida Levine, 1979 is a small group of Apicomplexa initially established for rhytidocystids—insufficiently investigated coccidian-like parasites of polychaetes^1^. Their life cycles are enigmatic. The Leuckart's triad (gametogony, merogony, and sporogony) typical of the sporozoan life cycle^2^ seems to be broken in rhytidocystids: no gametogony (sexual reproduction) and merogony (asexual reproduction) have been documented to date. Only sporozoites, large trophozoites embedded in the host intestinal epithelium, and coccidian-like oocysts with numerous sporocysts have been observed. Originally identified as gregarines from the family Monocystidae, rhytidocystids were later classified as coccidians^1,3,4^. Currently, the Agamococcidiorida comprises two families: Rhytidocystidae with five named species and Gemmocystidae with a single species *Gemmocystis cylindrus* originally described from eight species of Caribbean scleractinian corals. This parasite has been preliminarily assigned to agamococcidians because gamonts and other developmental stages beside sporozoites and oocysts (without sporocysts, unlike in *Rhytidocystis*) are not known^5^.

Until recently, only a couple of studies on molecular phylogeny of rhytidocystids have been published. Those phylogenies were based on 18S ribosomal RNA gene (rDNA) and did not conclusively resolve the rhytidocystid position^6,7^. No molecular data on *Gemmocystis* are available but, based on similarities in cell size, coccidian-like morphology, and localization in their hosts (mesenterial filaments), it was suggested that *G. cylindrus* belongs to corallicolids^8^. The corallicolids, previously called “genotype N” or “apicomplexans Type-N” in nuclear ribosomal DNA (rDNA) phylogenies^9,10^ and ARL-V in plastid rDNA phylogenies^11,12^, are related to eucoccidians^8,10^. In the most recent higher rank classification of protists^13^, the agamococcidians are assigned to the Apicomplexa *incertae sedis*. Lately, transcriptomes of two unclassified rhytidocystids were sequenced^14^, one of which corresponds to *Rhytidocystis pertsovi* sp. n. described in the present paper. The multiprotein phylogeny showed a basal position of rhytidocystids in the coccidiomorph clade^14^ but included only sparse species sampling without coralicollids and closest known relatives of *Rhytidocystis* such as *Margolisiella islandica*^15^. Clarifying the position of rhytidocystids in densely sampled phylogenies is therefore essential for the further development of apicomplexan systematics. In this study, we sequenced three new species of rhytidocystids from the White Sea and created phylogenies of Apicomplexa based on SSU rDNA alone and concatenated SSU, 5.8S, and LSU rDNAs. We here describe these new species as *Rh. nekhoroshkovae* sp. n. and *Rh. dobrovolskiji* sp. n. with light and scanning electron microscopy and *Rh. pertsovi* sp. n. with light, scanning and transmission electron microscopy. To assess whether the “asexual” rhytidocystids potentially retain the capacity for meiotic recombination, we surveyed the available transcriptomic data of *Rh. pertsovi* and yet undescribed *Rhytidocystis* sp. from *Travisia forbesii* for meiosis-specific genes. We also searched for the homologs of the coccidian oocyst wall proteins (OWPs) in rhytidocystids.

## Results

### Occurrence and morphology of new rhytidocystid species

#### Rhytidocystis nekhoroshkovae sp. n

Parasites were found in all 30 examined polychaetes *Pectinaria* (*Cistenides*) *hyperborea* collected in the vicinity of Educational and Research Station “Belomorskaya” of Saint Petersburg State University (ERS SPbU, see Methods for details). Infected midguts showed plenty of white dots on the outside surface, which corresponded to rhytidocystids, generally located at the basal part of the midgut epithelium. The polychaetes were usually heavily infected (hundreds of parasites per host).

**Figure 1:**
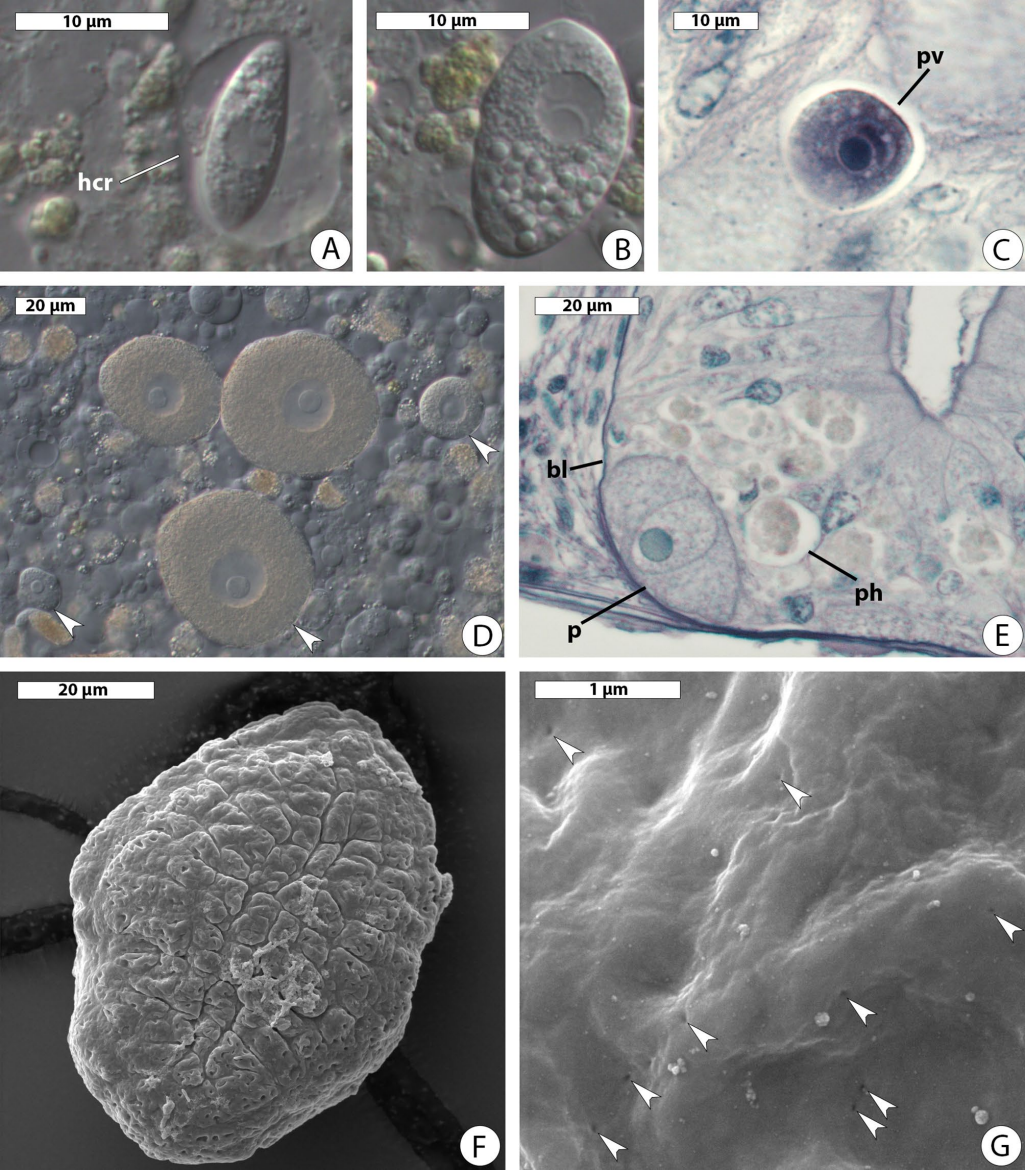
Morphology of *Rhytidocystis nekhoroshkovae* n. sp. (**A**) Spindle-shaped zoite on squash preparation of the host intestinal epithelium; *hcr* the host cell residue. DIC. (**B**) Growing zoite; squash preparation. DIC. (**C**) Histological section of *P. hyperborea* intestinal epithelium with young trophozoite inside parasitophorous vacuole (*pv*). LM. (**D**) Trophozoites (arrowheads) on squash preparation of the host intestinal epithelium. DIC. (**E**) Histological section of *P. hyperborea* intestinal epithelium with adult parasite (*p*); *bl* basal lamina, *ph* phagosome. LM. (**F**) Adult trophozoite. SEM. (**G**) Adult trophozoite’s cell surface with numerous micropores (arrowheads). SEM.

Spindle-shaped zoites were observed inside the host enterocytes (Fig. 1A). As zoites grew, they lost their elongated shape and transformed into trophozoites (Fig. 1B). Early development took place intracellularly and young trophozoites were located inside parasitophorous vacuoles (Fig. 1C). Both young and adult trophozoites had near-round or irregular shape with a slightly uneven border and measured 13.0–68.0 µm in maximal dimension (av. 47.4 ± 2.54 µm, n = 23). The trophozoites’ cytoplasm was filled with granules of storage carbohydrate (presumably, amylopectin), and smaller cells were more transparent than larger ones (Fig. 1D). Live parasites had a spherical nucleus located centrally and measured 6.6–19.2 µm in diameter (av. 14.64 ± 0.41 µm, n = 17). A single medium-sized spherical nucleolus was eccentric. Adult trophozoites were outside host cells close to the basal lamina of the midgut epithelium. No pathological changes were observed in infected tissue: the neighboring enterocytes had an appearance of active digestive cells with numerous phagosomes (Fig. 1E). Parasites isolated from the host gut were immotile. Their cell surface was rugose with longitudinal and transverse grooves, little creases, and small depressions (Fig. 1F). Numerous micropores on the parasite surface were arranged in curved rows, which merged with each other (Fig. 1G).

#### Rhytidocystis dobrovolskiji sp. n

Parasites were found in 34 out of 70 (48.6%) examined polychaetes *Ophelia limacina* collected in the vicinity of ERS SPbU (see Methods for details). Parasites were embedded in the host midgut epithelium and visible as white dots from the inside and outside the gut. The hosts contained between several and several dozens of parasites.

**Figure 2:**
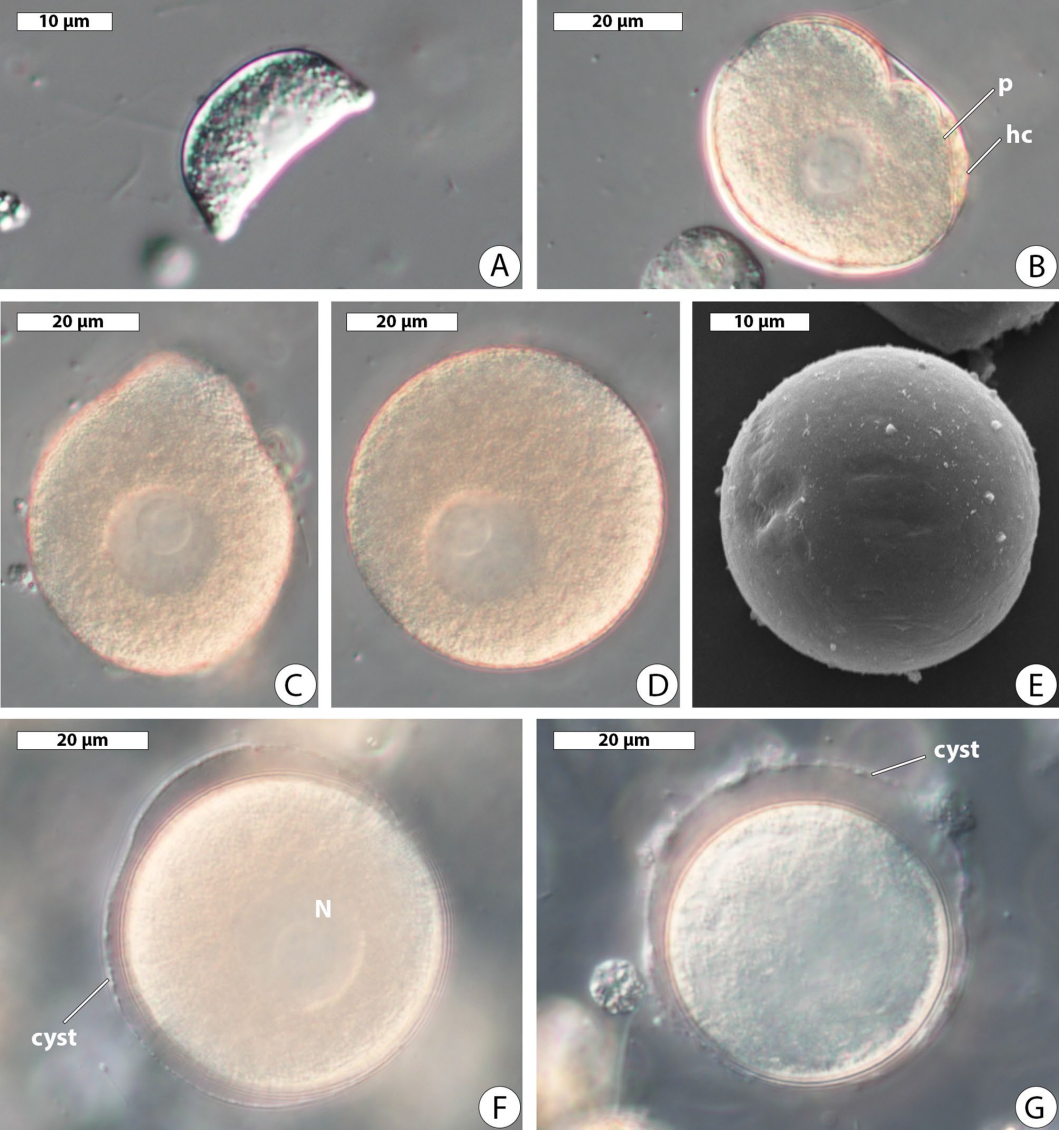
Morphology of *Rhytidocystis dobrovolskiji* n. sp. (**A**) Crescent-shaped trophozoite. DIC. (**B**) Tightly packed parasite (*p*) inside the host cell (*hc*). DIC. (**C**) Irregular trophozoite. DIC. (**D**) Spherical trophozoite. DIC. (**E**) Spherical trophozoite. SEM. (**F**,**G**) Young oocysts; *cyst* oocyst envelope, *N* nucleus. DIC.

The early trophozoite stages were crescent-shaped and measured 21.6–41.0 µm long (av. 32.9 ± 2.37 µm, n = 10) (Fig. 2A). Occasionally, trophozoites were found tightly packed inside the host cells (Fig. 2B). Maturing trophozoites measured 36.3–67.0 µm in maximal dimension (av. 51.0 ± 1.13 µm, n = 33), were irregular or roundish (Fig. 2C) and became spherical in a short time after the release from the host tissue (Fig. 2D). All forms had a spherical nucleus with a relatively large eccentric or centric nucleolus, usually located in the central part of the cell and measured 3.6–10.0 µm in diameter (av. 6.8 ± 0.61 µm, n = 10) in crescent-shaped forms and 12.1–25.0 µm (av. 18.2 ± 0.83 µm, n = 18) in maturing ones. The cell surface of spherical trophozoites was smooth (Fig. 2E). Only once we observed young oocysts released from the host intestinal epithelium. They were spherical and covered by a thick transparent envelope (Fig. 2F). The nuclei were not clearly visible in all of them (Fig. 2G).

#### Rhytidocystis pertsovi sp. n

Parasites were found in 73 out of 106 (68.9%) examined polychaetes *Ophelia limacina* collected at the White Sea Biological Station of Lomonosov Moscow State University (WSBS MSU, see Methods for details). Similar to *Rh. dobrovolskiji*, parasites were located in the host midgut epithelium. The infected polychaetes contained from a few up to hundreds of parasites.

**Figure 3:**
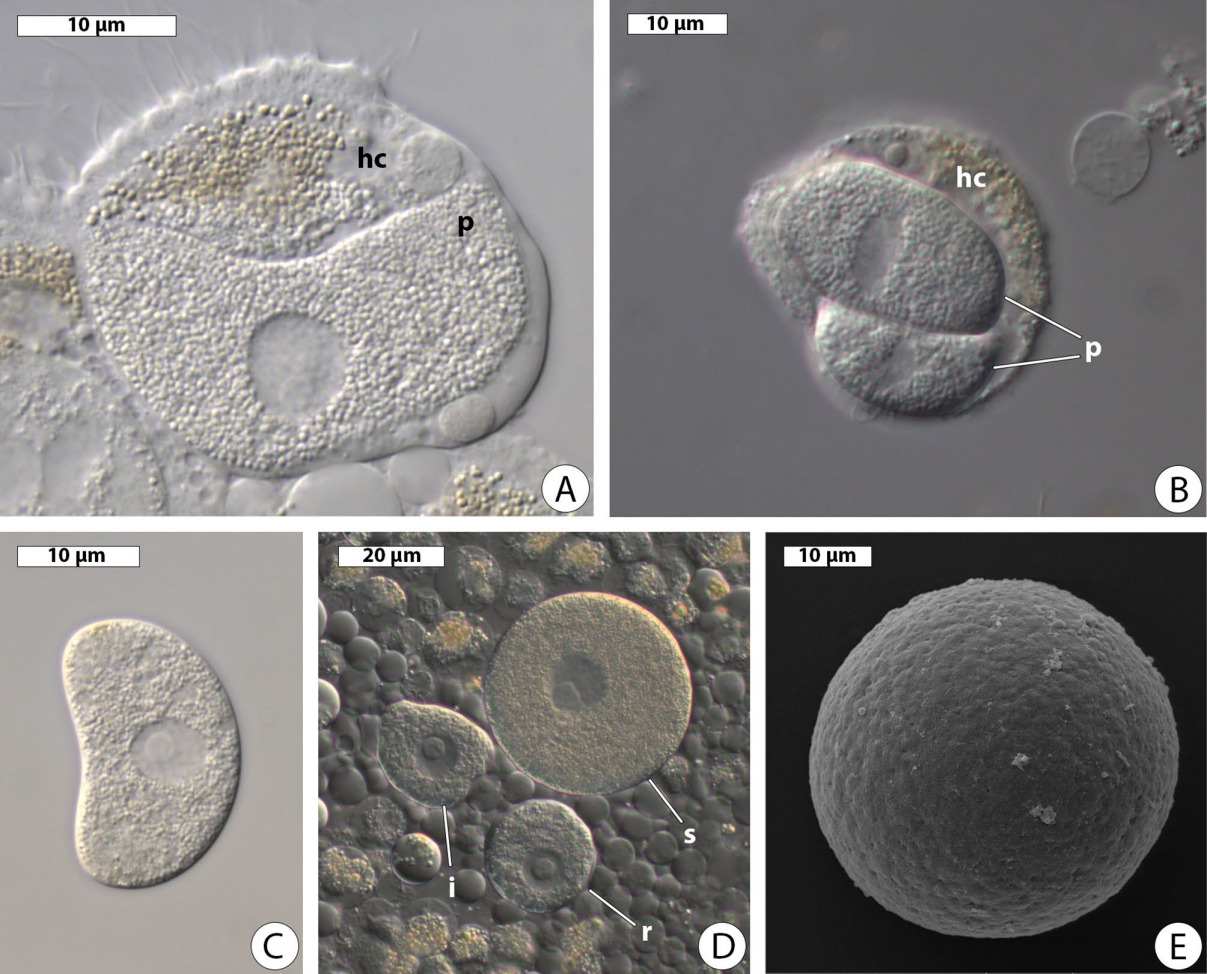
Morphology of *Rhytidocystis pertsovi* n. sp. (**A**) Host enterocyte with a crescent-shaped trophozoite inside; *hc* host cell, *p* parasite; squash preparation. DIC. (**B**) Host cell (*hc*) containing two parasites (*p*). DIC. (**C**) Bean-shaped trophozoite. DIC. (**D**) Squash preparation of the host intestinal epithelium with irregular (*i*), roundish (*r*) and spherical (*s*) trophozoites. DIC. (**E**) Spherical trophozoite. SEM.

Intracellular crescent-shaped trophozoites measured 14.3–25.3 µm long (av. 19.86 ± 1.36 µm, n = 7) were found inside the host enterocytes (Fig. 3A). Crescent-shaped trophozoites were sometimes located in pairs inside a one host cell (Fig. 3B). We observed several crescent-shaped trophozoites slowly becoming bean-shaped after the releasing from the host midgut epithelium (Fig. 3C). A spherical nucleus measuring 4.0–6.6 µm in diameter (av. 5.1 ± 0.51 µm, n = 5) was usually located in the central part of the cell and had a spherical eccentric nucleolus. Larger trophozoites, 21.3–59.2 µm in maximal dimension (av. 49.15 ± 1.66 µm, n = 32), were irregular or roundish in shape (Fig. 3D). They had a spherical centric nucleus measuring 8.0–20.0 µm in diameter (av. 15.79 ± 0.79 µm, n = 17) with a relatively large eccentric or centric nucleolus. Being released from the host tissue, they became spherical in a short time. The cell surface of spherical trophozoites had small depressions, but no folds (Fig. 2E).

**Figure 4:**
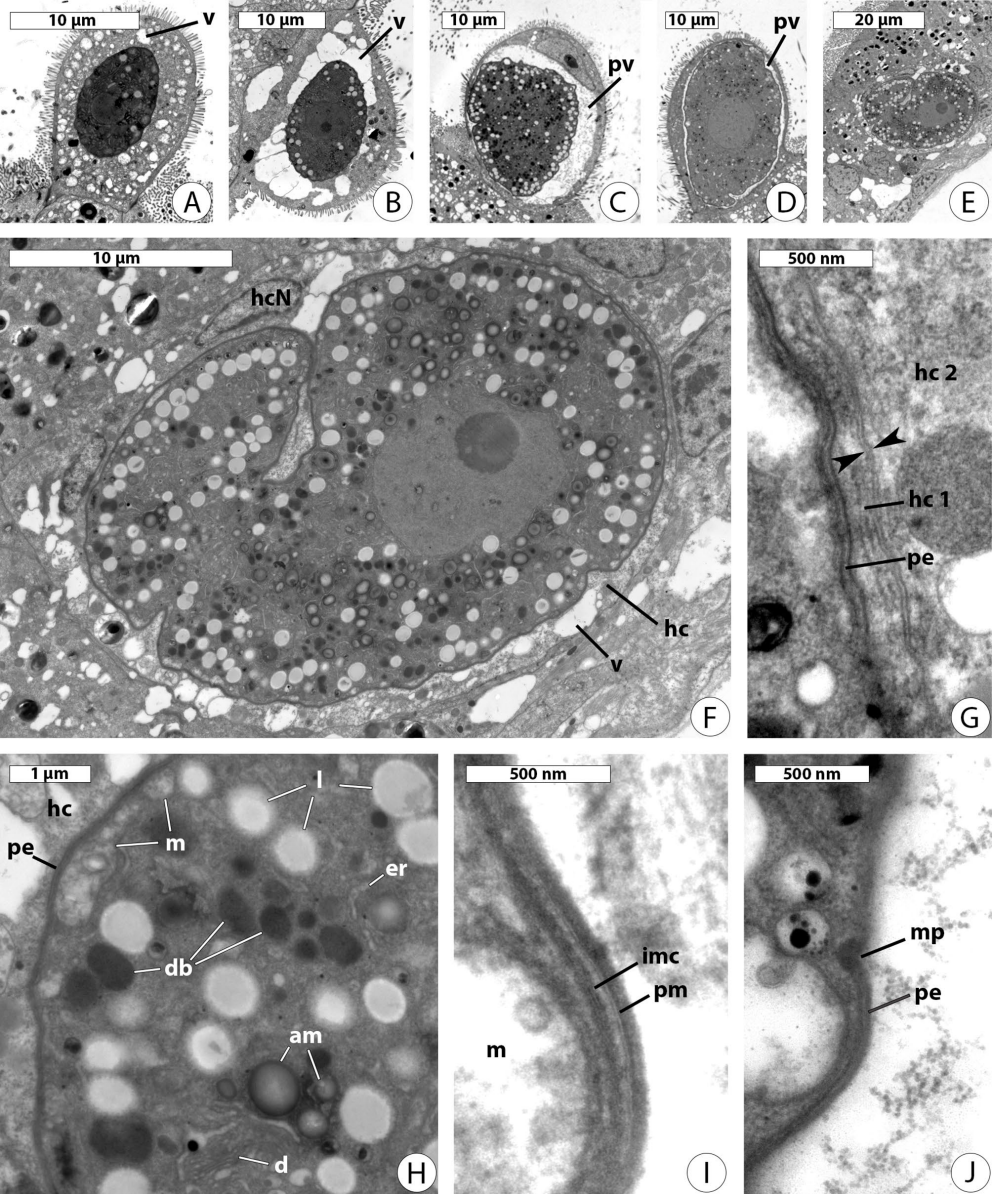
Transmission electron microscopy of intracellular stages *Rhytidocystis pertsovi* n. sp. (**A**–**D**) parasites in apical parts of enterocytes projected into the intestinal lumen. (**E**) Parasite in the middle of intestinal epithelium. (**F**–**H**) The same individual as in (**E**) under higher magnification; *hc* host cell, *hcN* host cell nucleus. (**I**,**J**) The same individual as in (**C**) under higher magnification. Parasites are in direct contact with the host cytoplasm (**A**,**B**,**E**,**F**) surrounded by small or large vacuoles (*v*) or inside a parasitophorous vacuole (*pv*) (**C**,**D**). (**G**) No parasitophorous vacuole membrane has been observed between the tegument of the parasite (the pellicle, *pe*) and adjacent plasma membranes (arrowheads) of two host cells: parasitized (hc 1) and neighboring (hc 2). (**H**) The trophozoite cytoplasm; *am* amylopectin granules, *d* dictyosomes, *db* dense bodies, *er* endoplasmic reticulum, *hc* host cell, *l* lipid droplets, *m* mitochondria, *pe* pellicle. (**I**,**J**) The trophozoite pellicle; *imc* inner membrane complex, *m* mitochondrion, *mp* micropore, *pe* pellicle, *pm* plasma membrane of parasite.

Transmission electron microscopy of the parasitized host intestine showed intracellular trophozoites both in the apical parts of enterocytes (Fig. 4A–D) and deep in the epithelium (Fig. 4E-F). Apical parts of infected enterocytes were extended into the intestinal lumen. Some parasites were in direct contact with the host cell cytoplasm (Fig. 4A,B,F,G) and surrounded by many small (Fig. 4A,F) or several large vacuoles (Fig. 4B). Other parasites were inside a parasitophorous vacuole (Fig. 4A,C). The general ultrastructure of the trophozoites was similar to that of intracellular coccidians (Fig. 4F–J). The tegument was represented by a trimembrane pellicle, which consisted of the plasma membrane and the inner membrane complex (Fig. 4I). Micropores were rarely present in the sections (Fig. 4J). The cytoplasm of trophozoites was filled with cisternae of the endoplasmic reticulum, dictyosomes, mitochondria having tubular cristae and mainly located under the pellicle, lipid droplets, granules of storage carbohydrate (probably, amylopectin) and numerous dense bodies of mostly oval or round shape and resembling oocyst wall forming bodies of some coccidians (Fig. 4H,I).

### Molecular phylogeny of *Rh*. *nekhoroshkovae*, *Rh*. *dobrovolskiji*, and *Rh*. *pertsovi*

The near-complete sequences of rRNA operon (SSU rDNA, ITS1, 5.8S rDNA, ITS2, and LSU rDNA) were obtained for *Rh. nekhoroshkovae* (5,410 bp), *Rh. dobrovolskiji* (5,387 bp), and *Rh. pertsovi* (5,281 bp). Despite parasitizing the same hosts and being very similar in appearance, *Rh. dobrovolskiji* and *Rh. pertsovi* were different genetically.

**Figure 5:**
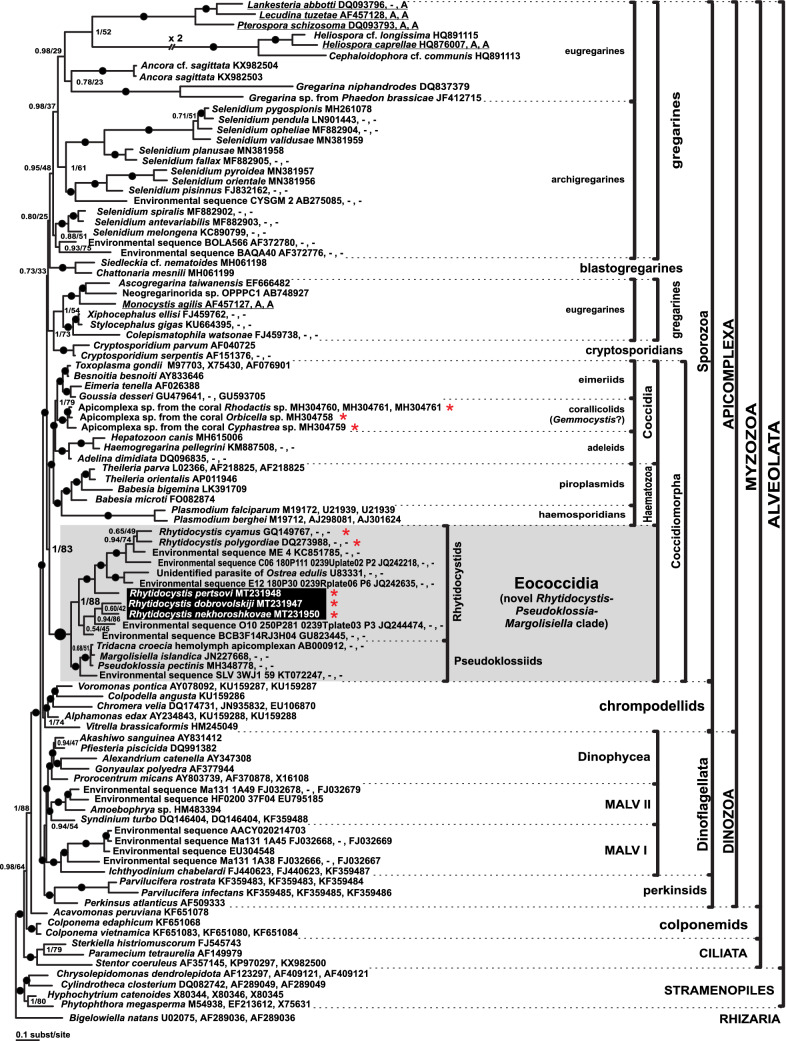
Bayesian inference tree of alveolates obtained by using the GTR + Г + I model from the dataset of 99 concatenated SSU, 5.8S, and LSU rDNA sequences (4,517 sites). Missing data on 5.8.S or/and LSU rDNA are marked by “–” in place of GenBank accessions; data assembled from transcriptomes are marked by “A”. Numbers at the nodes indicate Bayesian posterior probabilities (numerator) and ML bootstrap percentage (denominator). Black dots on the branches indicate Bayesian posterior probabilities and bootstrap percentages of 0.95 and 90% (respectively) and higher. The newly obtained sequences of *Rhytidocystis* spp. are on black background. The Eococcidia clade is highlighted by gray. Polyphyletic agamococcidians are marked by asterisks.

Both Bayesian (BI) and Maximum Likelihood (ML) analyses produced almost identical tree topologies except for the position of blastogregarines, which were either the sister lineage to coccidiomorphs (ML; not shown) or placed among gregarines (BI; Fig. 5). The Bayesian tree inferred from the concatenated rDNA dataset of 99 taxa and 4,517 sites (Fig. 5) showed the monophyly of major alveolate lineages with high posterior probabilities (PP), but with moderate or low ML supports (bootstrap percentages, BP) in the apicomplexan part of the tree.

The sequences of the three new *Rhytidocystis* species had relatively short branches and grouped into a common rhytidocystid clade with the other representatives of the genus (*Rh. cyamus* and *Rh*. *polygordiae*), an undescribed parasite of the European oyster *Ostrea edulis*, and several environmental sequences from oceanic sediments with PP = 1 and BP = 88%. The rhytidocystids then formed a robust higher-order clade (PP = 1.0 and BP = 100%) with an unidentified parasite of *Tridacna croecia*, the coccidians *Margolisiella islandica* and *Pseudoklossia pectinis*, and an environmental sequence from a sulfidic karst spring in Slovenia (KT072247). The robust *Rhytidocystis*-*Pseudoklossia-Margolisiella* clade was the most early-branching lineage of coccidiomorphs, including coccidians and haematozoans (PP = 1, BP = 83%). We suggested the name “Eococcidia” for this clade (Fig. 5, also see “Discussion”).

### Analysis of meiosis-specific and oocyst wall protein transcripts

**Figure 6:**
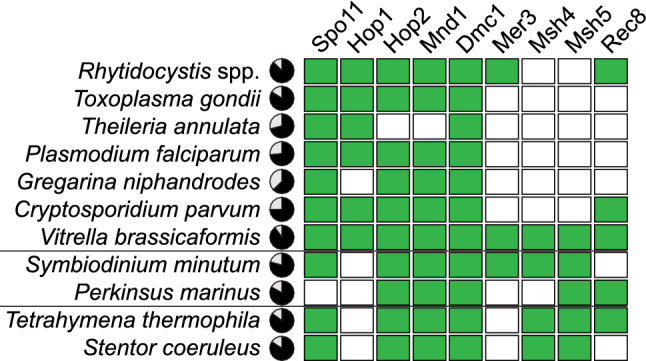
Occurrences of meiosis-specific genes in alveolates. *Rhytidocystis* spp. represents combined transcriptomic data of *Rh. pertsovi* and *Rhytidocystis* sp. ex *Travisia forbesii*. Pie charts show completeness scores of underlying genomic or transcriptomic data as estimated by BUSCO; filled boxes correspond to complete or fragmented orthologs. The Spo11 box includes either Spo11-1, Spo11-2, or both orthologs; non-meiotic Spo11-3/Top6A orthologs were not considered. Homologs reported in the Rec8 category include any findings of the Rad21/Rec8 family, as specific orthology of the proteins is difficult to determine (see also Supplementary Figs. S1–S9).

We examined the available transcriptomic data of rhytidocystids for transcripts of meiosis-specific genes to estimate whether meiotic recombination is possible in the reportedly asexual rhytidocystids. Homology searches identified seven meiosis-specific genes in the transcriptomic data of *Rh. pertsovi* and *Rhytidocystis* sp. ex *Travisia forbesii* (Fig. 6). Nearly all gene transcripts were partial or incompletely spliced in the assemblies, indicating low transcript presence in the sequencing libraries. Phylogenetic analyses grouped the rhytidocystid meiotic genes with other apicomplexans, ruling out that they could be contaminating sequences (Supplementary Figs. S1–S9). The rhytidocystid transcriptomes lack two genes of the core meiotic gene set—Msh4 and Msh5, but retain meiotic helicase Mer3 and a member of the Rad21/Rec8 cohesin family, which are both absent in sexual coccidians.

**Figure 7:**
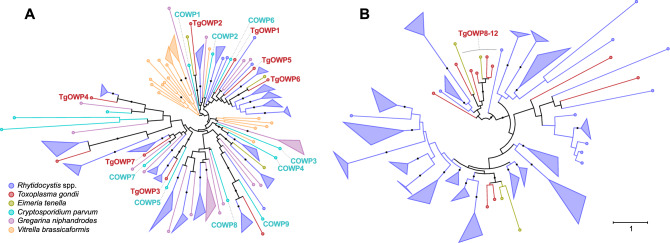
Phylogenetic reconstructions of oocyst wall proteins. (**A**) Maximum likelihood tree of COWP and TgOWP1-7 family homologs reconstructed with IQ-TREE (WAG + R5 evolutionary model selected by ModelFinder). Branches are colored according to the species legend (bottom left). Well-supported clusters (over 90% bootstrap support) of species-specific sequences are collapsed into triangles with side lengths proportional to the shortest and longest branches in the cluster. Branches with over 90% bootstrap support are marked with a black dot. The characterized OWPs of *Cryptosporidium* and *Toxoplasma* are labeled in the tree. *Rhytidocystis* spp. sequences represent homologs from the combined transcriptomic data of *Rhytidocystis pertsovi* and *Rhytidocystis* sp. ex *Travisia forbesii*. (**B**) Maximum likelihood tree of TgOWP8-12 family homologs reconstructed with IQ-TREE (DCMut + F + R5 evolutionary model selected by ModelFinder); all tree specifications are as in (**A**) (see also Supplementary Figs. S10,S11).

The discovery of young oocysts in *Rh. dobrovolskiji* and the presence of putative wall-forming bodies in *Rh. pertsovi* prompted us to search the rhytidocystid transcriptomes for genes encoding oocyst wall proteins previously described in *Toxoplasma* and *Cryptosporidium*. We found over one hundred transcripts related to the apicomplexan oocyst wall family proteins COWP and TgOWP1-7^16,17^ in the transcriptomes of the two *Rhytidocystis* species. Similarly to other apicomplexans, the rhytidocystid sequences are characterized by the N-terminal signal peptide followed by a series of cysteine-containing repeats. The tree reconstruction of apicomplexan COWP and TgOWP1-7 family proteins groups rhytidocystid sequences into 19 divergent clusters (Fig. 7A). The rhytidocystid OWP clusters are distributed evenly in the tree, pointing to their large diversity, with several clusters showing potential orthology to the characterized OWPs of *Cryptosporidium* and *Toxoplasma*: COWP4, COWP5, COWP9, TgOWP3, and TgOWP4 sequences. Another abundant family of candidate OWPs in the two rhytidocystid species (over 200 of total identified sequences) is homologous to the more recently described TgOWP8-12^18^. Similarly to the TgOWP1-7 family, the proteins contain periodical cysteine residues and a signal peptide. The TgOWP8-12 family was significantly expanded in rhytidocystids compared to the coccidians (Fig. 7B). However, that the majority of TgOWP1-7 and TgOWP8-12 homologs in the rhytidocystid transcriptomes are incomplete, which confounds their classification, especially considering the repetitive structure.

## Discussion

In the present study, we described three new species of *Rhytidocystis* agamococcidians, defined their phylogenetic position and surveyed for molecular markers of their sexual reproduction and oocyst wall formation. The new data reveal several key points on rhytidocystid biology and evolution.

### Rhytidocystids are most likely distributed worldwide and prefer to parasitize opheliid polychaetes

Previously described *Rhytidocystis* species were found on the Western and Eastern coasts of the North Atlantic and in the North-Eastern Pacific Ocean^3,4,6,7^. The rhytidocystids also parasitize polychaetes in the Arctic Ocean: the three new species described here come from the White Sea. In addition, environmental sequences belonging to the rhytidocystid clade (Fig. 5) were derived from the Yellow Sea (KC851785), the Caribbean Sea (GU823445) and the South Pacific (shallow water hydrothermal system near Papua New Guinea; JQ244474; JQ242635; JQ242218). Three out of the five previously described rhytidocystids parasitize polychaetes from the family Opheliidae: *Rh*. *opheliae*, *Rh*. *henneguyi,* and *Rh*. *cyamus*^3,4,7^. Here we described two new rhytidocystids from *Ophelia limacina*—*Rh*. *dobrovolskiji* and *Rh*. *pertsovi*. The third new species, *Rh. nekhoroshkovae*, parasitizes the polychaete from *Pectinaria*, the genus, which is relatively close to *Ophelia*^19,20^. Dissecting *O. verrilli*, Riser^21^ had also observed putative rhytidocystids (described as “coccidians”), which were located within projected apical ends of host enterocytes, similar to *Rh*. *pertsovi*. Apparently, most of the real biodiversity of rhytidocystids remains undiscovered.

### Eococcidia: the sister group of coccidians and haematozoans

Previous phylogenetic studies of SSU rDNA sequences did not resolve the position of rhytidocystids^6,7^, making it unclear whether they could be related to *Gemmocystis cylindrus*, the other agammococcidian now represented by the “genotype-N” and coralicollid sequences. In some phylogenies, *Rh. cyamus* and *Rh. polygordiae* were the sister group of the coccidians *Margolisiella islandica* and *Pseudoklossia pectinis*^15,22,23^, but their common clade was never strongly affiliated with gregarines, cryptosporidians, or coccidiomorphs. Reasons for the lack of resolution might be two-fold: *Rh. cyamus* and *Rh. polygordiae* form long tree branches which may cause the long branch attraction artifact, and the earlier studies used SSU rDNA phylogenies only, which have inferior resolution to the whole rRNA operon^24–27^. In the current study, we enlarged a broadly and evenly sampled alveolate dataset with environmental sequences and short-branching sequences of our new species. We also sequenced the first rhytidocystid LSU rDNA and analyzed their concatenated rDNA phylogeny in both Maximum likelihood and Bayesian frameworks. The resulting phylogenies have a resolution superior to earlier studies and resolve rhytidocystid and sporozoan relationships in two important ways. Firstly, rhytidocystids are unrelated to corallicolids which include *Gemmocystis cylindrus* (Fig. 5), demonstrating that the order Agamococcidiorida is polyphyletic. Secondly, the analyses unambiguously combined rhytidocystids with coccidiomorphs (coccidians and haematozoans). This finding matches the more sparsely-sampled but multiprotein phylogeny of apicomplexans based on 296 concatenated markers^14^, which recovered rhytidocystids as basal coccidiomorphs. *Margolisiella islandica* infecting the Iceland scallop *Chlamys islandica* is the closest described relative of rhytidocystids with complete Leukart’s triad in monoxenous life cycle (Fig. 5)^15,22,23^. *Pseudoklossia pectinis* is a putatively heteroxenous parasite of *Pecten maximus*: gamogony and sporogony occur in the great scallop, and a merogonic phase is supposed to be in some other host^28^. Other members of the *Rhytidocystis*-*Pseudoklossia-Margolisiella* clade are poorly-studied (unnamed parasites of *Tridacna croecia* and *Ostrea edulis*), and the whole clade lacks obvious shared morphological characteristics (synapomorphies). Nevertheless, since this robust clade has been recovered in several analyses and discovered as basal group of all coccidiomorphs, including coccidians and haematozoans, we suggest establishing the new taxon Eococcidia for basal coccidiomorphs mainly parasitizing marine invertebrates. The term “Eococcidia” refers to Eos—the goddess of the dawn in Greek mythology, who rose in the morning from the Oceanus; the prefix “eo-” is used in geology and biology for the designation of something to be early (Eococcidia—early coccidians). Coccidian genera *Merocystis* and *Aggregata* are also candidate members to Eococcidia since they were recovered as close relatives of *Rhytidocystis*, *Pseudoklossia,* and *Margolisiella*^23,29^.

### Intracellular parasitism links rhytidocystids with other coccidiomorphs

The phylum Apicomplexa contains both extracellular and intracellular parasites. Development of many gregarines runs extracellularly, whereas coccidians and haematozoans invade host cells^30,31^. Four out of five previously described rhytidocystids have trophozoites in the host intestinal epithelium^3,4,6,7^, but their intracellular stages were never detected. Here we provide evidence of intracellular localization of three new rhytidocystid species. The findings of putative sporozoites inside host cells and young trophozoites within parasitophorous vacuoles in *Rh. nekhoroshkovae*, and trophozoites tightly packed inside host cells in *Rh. dobrovolskiji* suggest that the development of both species begins intracellularly. The trophozoites of *Rh. pertsovi* on squash preparations of the host enterocytes and in TEM sections were undoubtedly located under the plasma membrane of the host cell. Perhaps, the development of other rhytidocystid may also start with intracellular forms. *Rh. polygordiae* was the only species previously described with TEM, and its trophozoites were observed in the interstitial space between adjacent host enterocytes^6^. However, the structure purported to be “the plasma membrane of the adjacent epithelial cell” (Fig. 19 in^6^) has the thickness (a little more than 20 nm) and appearance of two membranes with the intercellular space between them, and most likely represents two plasma membranes of two adjoining enterocytes. Furthermore, “the interstitial space” (Fig. 19 in^6^) is filled with structures resembling endoplasmic reticulum and apparently is the host cell cytoplasm. We therefore suppose that at least some trophozoites of *Rh. polygordiae* were located intracellularly. Overall, evidence for intracellular development strongly links rhytidocystids with other coccidiomorphs such as coccidians and haematozoans to the exclusion of gregarines and cryptosporidians, which are chiefly extracellular or epicellular. Congruent with their phylogeny (Fig. 5)^14^, and intracellular stages in *Margolisiella*^15^, this distribution suggests that intracellular invasion evolved in the common coccidiomorph ancestor. Unlike other coccidiomorphs, however, rhytidocystid trophozoites grow up to a relatively large size, destroy infected cells, and end up lying extracellularly within the host tissue.

Intracellular apicomplexans may be either embedded in a parasitophorous vacuole (PV) made of components of host origin or host and parasite origin, or be in direct contact with the host cell cytoplasm^32–34^. In the first case a zoite penetrates the host cell membrane, induces the PV formation and becomes surrounded by PV since the beginning of its intracellular development^35,36^. Unexpectedly, younger forms of *Rh. pertsovi* were not within a parasitophorous vacuole in our TEM sections (Fig. 4A,B), whereas several larger ones were (Fig. 4C,D). This result does not correspond to typical PV development, so a thorough TEM investigation of rhytidocystid intracellular development is needed.

### Morphology of trophozoite and oocyst stages

The trophozoite cell shape of earlier described *Rhytidocystis* species varies from oblong *Rh. polygordiae* and bean-shaped *Rh. cyamus* to flat oval cells of *Rh. opheliae* and *Rh. henneguyi*^3,4,6,7^. In the case of *Rh. pertsovi* and *Rh. dobrovolskiji*, it seems to be that crescent-shaped, bean-shaped, irregular, roundish and spherical forms represent successive stages of development. A zoite invades the host cell and transforms into a trophozoite. Presumably, during the growth inside the limited space of the host cell, the young trophozoite bends and becomes crescent-shaped, then it loses peaked cell poles and becomes bean-shaped; over time, it undergoes marked growth and becomes tightly packed under the host cell membrane. Released from the host cell to the interstitial space of the tissue, a trophozoite unbends, becomes irregular, then roundish and spherical eventually. Spherical trophozoites, apparently, represent the transitional form to the oocyst stage. Young oocysts *Rh*. *dobrovolskiji* look like spherical trophozoites covered by a thick transparent envelope and resemble young oocysts of *Rh. sthenelais*^37^.

The cytoplasm of intracellular trophozoites *Rh. pertsovi* looks typical of sporozoans and possesses all general structures. *Rh. pertsovi* retains an active apicoplast^14^ but none was observed in this TEM study. Instead, we found numerous dense bodies of oval or round shape, which could be homologous to wall-forming bodies (WFBs) in coccidian and cryptosporidian macrogamonts^38–41^. The WFBs mediate the formation of the oocyst wall, which more than 90% is made up of proteins^42^. The presence of oocysts and putative WFBs has prompted us to search in rhytidocystid transcriptomes for homologs of oocyst wall proteins (OWPs), some of which are proven to be located in WFBs^40,43^. The analysis revealed an astounding diversity of transcripts related to COWP, TgOWP1-7 and TgOWP8-12 family proteins, supporting the presence of WFBs in rhytidocystids and a common mechanism for their oocyst wall formation with coccidia and cryptosporidia.

### Discovery of meiotic genes in “asexual” rhytidocystids

Since rhytidocystids are closely related to *M. islandica,* which is an eucoccidian-like protist, whose life cycle includes all three types of sporozoan reproduction: gametogony, merogony, and sporogony (Leuckart's triad), and which, hence, produces sexual gamonts^15^, the supposed absence of sexual life stages in rhytidocystids raises the possibility that they recently lost sexual reproduction. Generally conserved across the eukaryotes, the core meiosis machinery includes nine proteins (Spo11, Hop1, Hop2, Mnd1, Dmc1, Mer3, Msh4, Msh5, Rec8), with functions spanning sister chromatid cohesion, induction of double-strand breaks, heteroduplex DNA and synaptonemal complex formation, and Holliday junction resolution^44,45^. In rhytidocystids, the core meiotic gene set is short of two genes for Msh4 and Msh5, unlike the chrompodellid *Vitrella brassicaformis*, which retains a full set of core meiosis-specific genes and where sexual process has been proposed^46^. The absence of the heterodimer-forming Msh4 and Msh5 in rhytidocystids is consistent with their absence in other coccidiomorphs’ genomes (Fig. 6): they are involved in the stabilization of Holliday junctions and meiotic crossover interference in model organisms^47^ but apparently dispensable in apicomplexans^48^. Notably, the closely related Msh2 family, which is involved in DNA repair, has expanded in *Rhytidocystis* sp. ex *Travisia forbesii*. Unlike many sporozoans, rhytidocystids retain the meiotic helicase Mer3 and a member of the Rad21/Rec8 cohesin family. Thus, the inventory of meiosis-specific genes in rhytidocystids does not display evidence of reduction in relation to the same gene set of sexual coccidians. The presence of these genes alone, however, does not constitute conclusive evidence of sexual reproduction in the family. Meiosis-specific genes, contrary to their designation, were reported to have functions outside of meiosis, specifically in homologous recombination and DNA repair^49^. The preservation of these genes weighs in favor of meiotic recombination in rhytidocystids, but more direct evidence would be necessary to verify the existence of sexual process.

In terms of appearance, rhytidocystid trophozoites are similar to macrogamonts of their closest relatives—sexual coccidians: they develop intracellularly, amass a supply of nutrients, produce wall-forming bodies and eventually become the oocysts. The presence of meiosis-specific transcripts challenges the long held belief that rhytidocystids lack gamogony and inspires search for their cryptic sexual process, which has remained hidden for over a hundred years. Future research on early rhytidocystid development and genetics are awaited to contribute to this matter.

### Taxonomic summary

Here we use the most recent Adl et al.^13^ system for higher-ranks as phylum and class, despite the inconsistency of this system to the actual phylogeny of Apicomplexa (Fig. 5)^14,26,50^, and due to the absence of a correct system. The eococcidians are as close to coccidians as to haematozoans (Aconoidasida) in our phylogeny. However, we classify Eococcidia into Conoidasida and Coccidia because of the findings of the apical complex in their zoites^6,37,51^. We suggest the order Pseudoklossiida for *Pseudoklossia* and *Margolisiella* (former Eimeriorina) as *P*. *pectinis* and *M. islandica* were recovered strongly within the eococcidians, but not within the eimeriids (Fig. 5)^15,23^. We keep the rhytidocystids into the order Agamococcidiida as no microgamonts and microgametes were found, only trophozoites that potentially may be macrogamonts. We do not include *Gemmocystis* to the Agamococcidiida as we consider it belonging to the corallicolids. The mature sporulated oocysts of the newly described rhytidocystids were not observed, but we use the characteristics of the previously described rhytidocystid oocysts for the taxon diagnosis^3,37^. We still lack distinct morphological synapomorphies both for Eococcidia and for Pseudoklossiida; therefore, the establishment of these taxa is mainly based on molecular data.

Phylum **Apicomplexa** Levine, 1970.

Class **Conoidasida** Levine, 1988.

Subclass **Coccidia** Leuckart, 1879.

Superorder **Eococcidia** superordo novus.

Coccidia. Homoxenous and heteroxenous parasites of marine invertebrates, predominantely polychaetes and mollusks. Molecular data: earlier robust sister clade to coccidians and haematozoans in rDNA and multiprotein phylogenies. Etymology: from “eo-”, a prefix meaning the earliest appearance, and “coccidia”.

Order **Pseudoklossiida** ordo novus.

Eococcidia. Homoxenous or heteroxenous parasites of marine molluscs (definitive hosts in heteroxenous); life cycle—complete Leuckart’s triad; development and merogony intracellular, sexuality intracellular or extracellular, fecundation intracellular or extracellular. *Margolisiella*, *Pseudoklossia*.

Order **Agamococcidiida** (Levine, 1979) emend.

Eococcidia. Merogony, microgamonts and microgametes not reported. *Rhytidocystis*.

Family **Rhytidocystidae** Levine, 1979.

Agamococcidiida. Early development intracellular; adult trophozoites extracellular in the host intestinal epithelium or coelom; large oocysts with many tens of sporocysts in the host intestinal epithelium or coelom; in annelids.

Genus ***Rhytidocystis*** Henneguy, 1907[= Dehornia Porchet-Henneré, 1972].

***Rhytidocystis nekhoroshkovae*** **sp. n.** Miroliubova, Simdyanov, Janouškovec, Paskerova, 2020.

*Description*. Trophozoites 13.0–68.0 µm, flattened, almost round or irregular; immotile; young stages intracellular, adults extracellular. Spherical centric nucleus 6.6–19.2 µm with a spherical centric nucleolus. Cell surface of adult trophozoites rugose with longitudinal and transverse grooves, little creases and small depressions and numerous micropores in the curved rows, which merged with each other.

*Molecular data*. Partial rDNA (SSU rDNA, ITS1, 5.8S rDNA, ITS2, and LSU rDNA), GenBank accession number MT231950.

*Type locality*. White Sea, Kandalaksha Gulf, Chupa Bay, Podpakhta Strait (66.301129 N, 33.622062 E), a depth of ~ 20 m.

*Type habitat*. Marine.

*Type host*. *Pectinaria* (*Cistenides*) *hyperborea* Malmgren, 1866 (Polychaeta: Pectinariidae).

*Location in host*. Midgut epithelium.

*Type (syntype) material*. A platinum sputter-coated SEM stub with several protists, slides with histological sections, specimen of parasite cells and host material fixed in ethanol have been deposited in the collection of Department of Invertebrate Zoology, Saint Petersburg State University; extracted DNA used for obtaining of rDNA sequences deposited in the collection of Department of evolutionary biochemistry, Belozersky Institute for Physico-Chemical Biology, Lomonosov Moscow State University; Fig. 1 (this publication) shows some of the syntypes.

### *Zoobank registration. LSID* urn:lsid:zoobank.org:act:A80C2D4C-238E-4E30-A6D5-E43DF2D379A2

*Etymology*. This species was named in honor of Svetlana Nekhoroschkova, PhD, an inspiring ecology lecturer in the Ecological and Biological Lyceum (Arkhangelsk, Russia), dear teacher of the author Tatiana Miroliubova.

***Rhytidocystis dobrovolskiji*** **sp. n.** Miroliubova, Simdyanov, Janouškovec, Paskerova, 2020.

*Description*. Young intracellular trophozoites 21.6–41.0 µm, crescent-shaped. Larger trophozoites 36.3–67.0 µm; irregular, roundish then spherical and smooth; extracellular. Spherical centric nucleus with relatively big eccentric or centric nucleolus 3.6–10.0 µm in young and 12.1–25.0 µm in larger forms. Young oocyst within thick transparent envelope.

*Molecular data*. Partial rDNA (SSU rDNA, ITS1, 5.8S rDNA, ITS2, and LSU rDNA), GenBank accession number MT231947.

*Type locality*. White Sea, Kandalaksha Gulf, Chupa Bay, Yakovleva Inlet (66.315649 N, 33.836669 E) at a depth of ~ 1–15 m.

*Type habitat*. Marine.

*Type host*. *Ophelia limacina* Rathke, 1843 (Polychaeta: Opheliidae).

*Location in host*. The midgut epithelium.

*Type (syntype) material*. A platinum sputter-coated SEM stub with several protists, specimen of parasite cells and host material fixed in ethanol have been deposited in the collection of Department of Invertebrate Zoology, Saint Petersburg State University; extracted DNA used for obtaining of rDNA sequences deposited in the collection of Department of evolutionary biochemistry, Belozersky Institute for Physico-Chemical Biology, Lomonosov Moscow State University; Fig. 2 (this publication) shows some of the syntypes.

### *Zoobank registration. LSID* urn:lsid:zoobank.org:act:6B80FBE6-63E7-4CCF-A054-8D76E142320E

*Etymology*. This species was named after Dr Andrej Alexandrovitch Dobrovolskij (1939–2019), Professor of the Department of Invertebrate Zoology at Saint Petersburg State University, a recognized authority in the field of Invertebrate Zoology and Parasitology, the Great Teacher of many generations of Russian zoologists, who devoted himself utterly to science and students. In particular, he taught and inspired the authors TSM, TGS, and GGP.

***Rhytidocystis pertsovi*** **sp. n.** Miroliubova, Simdyanov, Janouškovec, Belova, Paskerova, 2020.

*Description*. Young intracellular trophozoites 14.3–25.3 µm; crescent-shaped then bean-shaped with a spherical centric nucleus 4–6.6.0 µm and a spherical eccentric nucleolus. Larger trophozoites extracellular, 21.3–59.2 µm; irregular, roundish then spherical; with a spherical centric nucleus 8.0–20.0 µm and a relatively big eccentric or centric nucleolus. The cell surface of spherical trophozoites has no folds.

*Molecular data*. Partial rDNA (SSU rDNA, ITS1, 5.8S rDNA, ITS2, and LSU rDNA), GenBank accession number MT231948; transcriptome shotgun assembly, GenBank accession number GHVQ00000000.1.

*Type locality*. White Sea, Kandalaksha Gulf (66.556758 N, 33.106862 E), a depth of ~ 1–15 m.

*Type habitat*. Marine.

*Type host*. *Ophelia limacina* Rathke, 1843 (Polychaeta: Opheliidae).

*Location in host*. The midgut epithelium.

*Type (syntype) material*. A platinum sputter-coated SEM stub with several protists, specimen of parasite cells and host material fixed in ethanol have been deposited in the collection of Department of Invertebrate Zoology, Saint Petersburg State University. Resin blocks and fixed slides containing pieces of infected host intestine deposited in the collection of the author TGS, Department of Invertebrate Zoology, Lomonosov Moscow State University; extracted DNA used for obtaining of rDNA sequences deposited in the collection of Department of evolutionary biochemistry, Belozersky Institute for Physico-Chemical Biology, Lomonosov Moscow State University; Figs. 3, 4 (this publication) show some of the syntypes.

### *Zoobank registration LSID.* urn:lsid:zoobank.org:act:71B99A74-6B81-40C0-87CA-5D0FF8D33F39

*Etymology.* This species was named after Nickolay Pertsov (1924–1987), a long-time director and an eminent innovator at the White Sea Biological Station of Lomonosov Moscow State University, where this species was found and sampled.

## Materials and methods

Polychaete hosts were collected from the sublittoral zone in different sites in Kandalaksha Gulf, White Sea in 2015–2018. The bristle worms *Pectinaria* (*Cistenides*) *hyperborea* Malmgren, 1866 (Polychaeta: Pectinariidae) were found in the vicinity of Educational and Research station “Belomorskaya” of the Saint Petersburg State University (ERS SPbU), Chupa Bay, Podpakhta Strait (66.301129 N, 33.622062 E), at a depth of ~ 20 m, under the thermocline. Bristle worms *Ophelia limacina* Rathke, 1843 (Polychaeta: Ophellidae) were collected from two distant locations: White Sea Biological Station Lomonosov Moscow State University (WSBS MSU), Velikaja Salma, Kandalaksha Bay, White Sea (66.556758 N, 33.106862 E) and in the vicinity of ERS SPbU, Yakovleva Inlet, Keret’ Archipelago, Chupa Bay, Kandalaksha Bay, White Sea (66.315649 N, 33.836669 E) at a depth of ~ 1–15 m. The *O. limacina* worms from these different locations were stored and processed independently. The coccidian individuals and pieces of the infected host intestine were isolated with fine tip needles under Olympus SZ40 (Olympus, Japan) or MBS-10 (LOMO, Russia) stereomicroscopes.

### Light microscopy

Fragments of the infected host intestine were fixed with Bouin solution, rinsed two times with distilled water and dehydrated in ethanol series. Using paraffin-celloidin method^52^ 4 μm thick sections were made with the Leica RM-2265 microtome and stained with Ehrlich's Hematoxylin. Separate alive parasites and squash preparations of the host intestine fragments containing parasites as well as histological sections were photographed with the help of Leica DM 2500 light microscopes equipped with differential interference contrast (DIC) optics and Plan-Apo objective lenses and connected to a Leica DFC295 or a Nikon DS-Fi1 digital cameras (Leica Microsystems, Germany; Nikon Corporation, Japan). All in vivo microphotographs were taken with the use of DIC technique, microphotographs of histological sections—by means of bright-field microscopy (LM). Maximal dimensions of protist cells were measured with the ImageJ program (rsb.info.nih.gov/ij/). Average values and standard errors were calculated.

### Electron microscopy

The cell surface of the isolated parasites was studied with scanning electron microscopy (SEM). Trophozoites *Rhytidocystis pertsovi* were also studied with transmission electron microscopy (TEM). For both methods, the individual trophozoites or small fragments of the infected host intestine were fixed with 2.5% (v/v) glutaraldehyde in 0.05 M cacodylate buffer (pH 7.4) containing 1.28% (w/v) NaCl in an ice bath in the dark. The fixative was once replaced with fresh fixative after 1 h, and the total fixation time was 2 h. The fixed samples were rinsed three times with cacodylate buffer and post-fixed with 2% (w/v) osmium tetroxide in the cacodylate buffer (ice bath, 2 h). After fixation, samples were dehydrated in an ethanol series.

For SEM dehydrated samples were critical point dried in liquid CO2 and then sputter-coated with platinum. The samples were investigated with a Tescan MIRA3 LMU scanning electron microscope (TESCAN, Czech Republic), and FEI Quanta 250 (Thermo Fisher Scientific, Netherlands).

For TEM study dehydrated samples were transferred to an ethanol/acetone mixture 1:1 (v/v), rinsed twice in pure acetone, and embedded in Epon resin using a standard procedure. Ultrathin sections obtained using LKB-III (LKB-produkter, Sweden) or Leica EM UC6 (Leica Microsystems, Germany) ultramicrotomes were contrasted with uranyl acetate and lead citrate^53^ and examined under a JEM 1011 electron microscope (JEOL, Tokyo, Japan).

### DNA extraction, PCR amplification, and sequencing of rDNA

Isolated trophozoites (up to 50 cells from each location) were washed three times in filtered sea water and deposited into 1.5 ml microcentrifuge tubes. Samples of *Rh. nekhoroshkovae* and *Rh. pertsovi* were fixed with 96% ethanol, the sample of *Rh. dobrovolskiji* was fixed with RNA-later (Life Technologies, USA). Extraction of DNA from fixed cells was performed using the NucleoSpin Tissue kit (Macherey–Nagel GmbH & Co. KG, Germany). Whole Genome Amplification (WGA) was performed for *Rh. nekhoroshkovae* and *Rh. dobrovolskiji* samples using REPLI-g Midi Kit (Qiagen, UK). The contiguous nucleotide sequences (SSU, ITS1, 5.8S, ITS2 and LSU rDNAs) were assembled from a series of overlapping fragments obtained by PCR with different pairs of primers, followed by Sanger sequencing (see^26,27,50^ for the general approach). The rDNA fragments were amplified with Encyclo PCR kit (Evrogen, Russia) in a total volume of 20 µl using a DNA Engine Dyad thermocycler (Bio-Rad) and a T100 Thermal Cycler (Bio-Rad). General scheme of PCR protocol as follows: lid temperature 100 °C; initial denaturation at 95 °C for 2.5 min; 40 cycles of 95 °C for 30 s (denaturation); 48–55 °C for 30 s (annealing); 72 °C for 1.5 (elongation) and a final extension at 72 °C for 10 min. Table 1 shows the lengths of amplified and overlapping fragments, sequences of used oligonucleotides and exact annealing temperatures.

**Table 1:** Main characteristics of the rhytidocystid sequences obtained in this study.

Object, length of contig, and its accession number	Amplified fragment	Length	Overlap	Primers: forward (F) and reverse (R); annealing temperature used in the PCRs
*Rhytidocystis nekhoroshkovae* 5,410 bp MT231950	(I) SSU rDNA (part)	~ 1,780 bp	98 bp	(F) ͣ 5′-GTATCTGGTTGATCCTGCCAGT-3’ (R) 5′-GGAAACCTTGTTACGACTTCTC-3’ t° = 48 °C
	(II) SSU rDNA (part), ITS1, 5.8S rDNA, ITS2, LSU rDNA (part)	~ 1,200 bp	619 bp	(F) 5′-GTACACACCGCCCGTCGCTC-3’ (R) 5′-CCTTGGTCCGTGTTTCAAGAC-3’ t° = 50 °C
	(III) LSU rDNA (part)	~ 2,170 bp	892 bp	(F)^b^ 5′-ACCCGCTGAAYTTAAGCATAT-3’ (R)^b^ 5′-ACATTCAGAGCACTGGGCAG-3’ t° = 50 °C
	(IV) LSU rDNA (part)	~ 1,350 bp	397 bp	(F)^b^ 5′-TCCGCTAAGGAGTGTGTAACAAC-3’ (R)^b^ 5′-CCGCCCCAGYCAAACTCCC-3′ t° = 53 °C
	(V) LSU rDNA (part)	~ 1,090 bp		(F)^b^ 5′-GATTTCTGCCCAGTGCTCTG-3′ (R)^b^ 5′-MRGGCTKAATCTCARYRGATCG-3′ t° = 55 °C
*Rhytidocystis dobrovolskiji* 5,387 bp MT231947	(I) SSU rDNA (part)	~ 1,760 bp	63 bp	(F) 5′-TMYCYGRTTGATYCTGYC-3′ (R) 5′-GGAAACCTTGTTACGACTTCTC-3′ t° = 48 °C
	(II) SSU rDNA (part), ITS1, 5.8S rDNA, ITS2, LSU rDNA (part)	~ 1,230 bp	647 bp	(F)^c^ 5′-GGTCCGGTGAATTAACCAGATT-3′ (R)^c^ 5′-CCTTGGTCCGTGTTTCAAGAC-3′ t° = 50 °C
	(III) LSU rDNA (part)	~ 1,950 bp	636 bp	(F)^b^ 5′-ACCCGCTGAAYTTAAGCATAT-3′ (R)^b^ 5′-AGCCAATCCTTWTCCCGAAGTTAC-3′ t° = 53 °C
	(IV) LSU rDNA (part)	~ 1,350 bp	∼395 bp	(F)^b^ 5′-TCCGCTAAGGAGTGTGTAACAAC-3′ (R)^b^ 5′-CCGCCCCAGYCAAACTCCC-3′ t° = 53 °C
	(V) LSU rDNA (part)	~ 1,010 bp		(F)^b^ 5′-GATTTCTGCCCAGTGCTCTG-3′ (R)^b^ 5′-MRGGCTKAATCTCARYRGATCG-3′ t° = 55 °C
*Rhytidocystis pertsovi* 5,281 bp MT231948	(I) SSU rDNA (part)	~ 1,680 bp	101 bp	(F) 5′-TMYCYGRTTGATYCTGYC-3′ (R) 5′-GGAAACCTTGTTACGACTTCTC-3′ t° = 48 °C
	(II) SSU rDNA (part), ITS1, 5.8S rDNA, ITS2, LSU rDNA (part)	~ 1,170 bp	601 bp	(F) 5′-GTACACACCGCCCGTCGC-3′ (R) 5′-CCTTGGTCCGTGTTTCAAGAC-3′ t° = 50 °C
	(III) LSU rDNA (part)	~ 2,150 bp	891 bp	(F)^b^ 5′-ACCCGCTGAAYTTAAGCATAT-3′ (R)^b^ 5′-ACATTCAGAGCACTGGGCAG-3′ t° = 50 °C
	(IV) LSU rDNA (part)	~ 2,020 bp		(F)^b^ 5′-TCCGCTAAGGAGTGTGTAACAAC-3′ (R)^b^ 5′-MRGGCTKAATCTCARYRGATCG-3′ t° = 53 °C

^a^The primer sequence was based on Medlin et al.^54^.

^b^The primer sequences were based on Van der Auwera et al.^55^.

^b^The primer sequence was tailored specially for *Rh. dobrovolskiji.*

### Assembling of rRNAs sequences from transcriptomic data

The rDNAs of *Pterospora schizosoma*, *Monocystis agilis*, *Lecudina tuzetae*, and *Heliospora caprellae* were assembled from the transcriptome sequencing data generated by Mathur et al.^56^. The rDNAs of *Lankesteria abbotti* were assembled from the transcriptome sequencing data of The Marine Microbial Eukaryote Transcriptome Sequencing Project^57^. The sequencing data were obtained from the NCBI Sequence Read Archive (https://www.ncbi.nlm.nih.gov/sra; accessions: SRR8980200-SRR8980205, SRR8980208-SRR8980213, SRR1300212), and the assemblies were performed with SPAdes^58^ utilizing k-mer size 127 and Trinity^59^ programs. Assembled rDNA contigs were aligned using MAFFT^60^ and inspected by eye for assembly errors and chimeric sequences.

### Molecular phylogenetic analyses

The alignment of concatenated SSU, 5.8S, and LSU rDNAs (54 sequences, 4,618 sites) was prepared for phylogenetic analyses. The taxon sampling was designed in order to maximize the phylogenetic diversity of Apicomplexa and completeness of sequences in alignments, by preferentially selecting taxa having both SSU and LSU rDNA sequences. However, since the taxon samplings of 5.8S and LSU rDNAs are limited by available sequences, we then expanded the dataset by adding SSU rDNA-alone sequences of previously published *Rhytidocystis* spp., their closest relatives (several environmental sequences, *Pseudoklossia pectinis* and *Margolisiella islandica*), two adeleid coccidians (additionally to *Hepatozoon canis*), and major gregarine lineages not represented in 5.8S and LSU rDNA databases (Stylocephaloidea eugregarines and archigregarine lineage IV from terebellid polychaetes). The missing nucleotide sites of 5.8S and LSU rDNAs were marked as "N" in the concatenated dataset. The rDNAs' alignments were generated in MUSCLE 3.6^61^ under default parameters and then manually adjusted and concatenated with BioEdit 7.0.9.0^62^; columns containing few nucleotides or hypervariable regions were removed. Representatives of stramenopiles and Rhizaria were used as outgroups. The final dataset included 99 taxa with 4,517 unambiguously aligned sites.

Maximum-likelihood (ML) analyses were performed with RAxML 8.2.9^63^ under the GTR + Г model and CAT approximation (25 rate categories per site). The procedure included 100 alternative runs of the ML analysis and 1,000 replicates of multiparametric bootstrap. Bootstrap percentages were merged on the user trees (both ML and BI) with the same program. Bayesian inference (BI) analyses were done in MrBayes 3.2.6^64^ under GTR + Г + I model with 12 discrete categories of gamma distribution. The following parameters were used: nst = 6, ngammacat = 8, rates = invgamma; parameters of Metropolis Coupling Markov Chains Monte Carlo (mcmc): nruns = 2, nchains = 4, temp = 0.2, ngen = 10,000,000, samplefreq = 1,000, burninfrac = 0.5. The average standard deviation of split frequencies at the end of computations was 0.001441.

### Analysis of meiosis-specific and oocyst wall protein transcripts

Searches for meiosis-specific and oocyst wall protein families in the transcriptomes of *Rhytidocystis* species were carried out with HMMER^65^ using profiles constructed from protein family alignments. The corresponding protein families were identified with OrthoFinder^66^ clustering with predicted protein sequences in a set of 70 eukaryotic genomes; the protein family alignments were generated using MAFFT^60^. We utilized the following sources for genomic data: Genome database of NCBI (https://www.ncbi.nlm.nih.gov/genome), Genome Portal of DOE JGI (https://genome.jgi.doe.gov/portal/), Ensembl Protists resources (https://protists.ensembl.org/), genome projects of Marine Genomics Unit (https://marinegenomics.oist.jp/), and genomic resources of multicellgenome lab (https://multicellgenome.com/). The transcriptomic data for *Rhytidocystis pertsovi* and *Rhytidocystis* sp. ex *Travisia forbesii* were obtained from GenBank transcriptome shotgun assembly projects GHVQ00000000.1 and GHVS00000000.1. The transcriptome assemblies of *Rhytidocystis* species were processed with TransDecoder^67^ utilizing BLAST^68^ and HMMER searches against the UniProtKB/Swiss-Prot^69^ and Pfam^70^ databases for ORF prediction. Orthology of proteins discovered by HMMER profile searches was verified by reciprocal BLAST searches against OrthoFinder orthogroups: proteins with best hit outside of the queried protein family were excluded from the set of findings. The findings satisfying reciprocal BLAST search criterion were added to the protein family alignments using MAFFT^60^, and the family membership was further inspected by reconstructing phylogenies. The trees for meiosis-specific protein families were reconstructed by IQ-TREE^71^ using the LG + C10 + F + G4 profile mixture model, and ultrafast bootstrap approximation^72^ with 1,000 replicates for estimation of branch support; IQ-TREE reconstructions for oocyst wall protein families utilized ModelFinder^73^ to automatically select the best-fit model. The trees were visualized using MEGA^74^ and iTOL^75^. The completeness estimates for genomic and transcriptomic data were performed with BUSCO^76^ using the eukaryota_odb9 dataset.

## Supplementary information

Supplementary file1
