# Supplementary material for: Polyphyletic origin, intracellular invasion, and meiotic genes in the putatively asexual agamococcidians (Apicomplexa incertae sedis)
Source: Sci Rep. 2020 Sep 28;10:15847. doi: 10.1038/s41598-020-72287-x (PMC7522995; doi:10.1038/s41598-020-72287-x)
Supplement: Supplementary file 1 — Supplementary file1 [file 41598_2020_72287_MOESM1_ESM.pdf]

Polyphyletic origin, intracellular invasion, and meiotic genes in the putatively asexual agamococcidians (Apicomplexa *incertae sedis*)

Tatiana S. Miroliubova[1,2*], Timur G. Simdyanov[3], Kirill V. Mikhailov[4,5], Vladimir V. Aleoshin[4,5], Jan Janouškovec[6], Polina A. Belova[3], Gita G. Paskerova[2]

[1]Severtsov Institute of Ecology and Evolution, Russian Academy of Sciences, Leninsky pr. 33, Moscow, 117071, Russian Federation; [2]Department of Invertebrate Zoology, Faculty of Biology, Saint Petersburg State University, Universitetskaya emb. 7/9, 199034, Saint Petersburg, Russian Federation; [3]Faculty of Biology, Lomonosov Moscow State University, ul. Leninskiye Gory, 1c12, 119991, Moscow, Russian Federation; [4]Belozersky Institute for Physico-Chemical Biology, Lomonosov Moscow State University, ul. Leninskiye Gory, 1c40, 119992, Moscow, Russian Federation; [5]Kharkevich Institute for Information Transmission Problems, Russian Academy of Sciences, Bolshoy Karetny per. 19c1, 127051, Moscow, Russian Federation; [6]University of Oslo, Department of Pharmacy, Sem Sælands vei 2C, 0371 Oslo, Norway.
*email: provorosenok@gmail.com

Supplementary Figures S1-S9. Maximum likelihood trees reconstructed by IQ-TREE (LG+C10+F+G4 model) for meiosis-specific protein families: (**S1**) Spo11, (**S2**) Hop1, (**S3**) Hop2, (**S4**) Mnd1, (**S5**) Dmc1, (**S6**) Mer3, (**S7**) Msh4, (**S8**) Msh5, (**S9**) Rad21/Rec8. Branch support was estimated by ultrafast bootstrap approximation with 1000 replicates. The alveolate sequences in the trees are marked with blue color, while likely contaminating sequences discovered in the corresponding transcriptomic data are marked with red color; the rhytidocystid sequences are highlighted yellow. Accessions are provided next to the sequence names in lighter color; sequence read archive (SRA) accessions are given for sequences acquired from assemblies of raw sequencing data; the MMETSP type accessions correspond to the transcript data of the Marine Microbial Eukaryotic Transcriptome Sequencing Project re-assembly dataset (https://zenodo.org/record/257026).

Supplementary Figure S10. Maximum likelihood tree of COWP-TgOWP1-7 family proteins reconstructed with IQ-TREE (WAG+R5 evolutionary model selected by ModelFinder); branch support was estimated by ultrafast bootstrap approximation with 1000 replicates. The rhytidocystid sequences are labeled with blue color; sequence accessions are written next to the sequence names in lighter color; the characterized sequences of *Cryptosporidium* and *Toxoplasma* OWPs are shown in the tree with the corresponding aliases.

Supplementary Figure S11. Maximum likelihood tree of TgOWP8-12 family proteins reconstructed with IQ-TREE (DCMut+F+R5 evolutionary model selected by ModelFinder); branch support was estimated by ultrafast bootstrap approximation with 1000 replicates. The rhytidocystid sequences are labeled with blue color; sequence accessions are written next to the sequence names in lighter color; the characterized sequences of *Toxoplasma* OWPs are outlined.

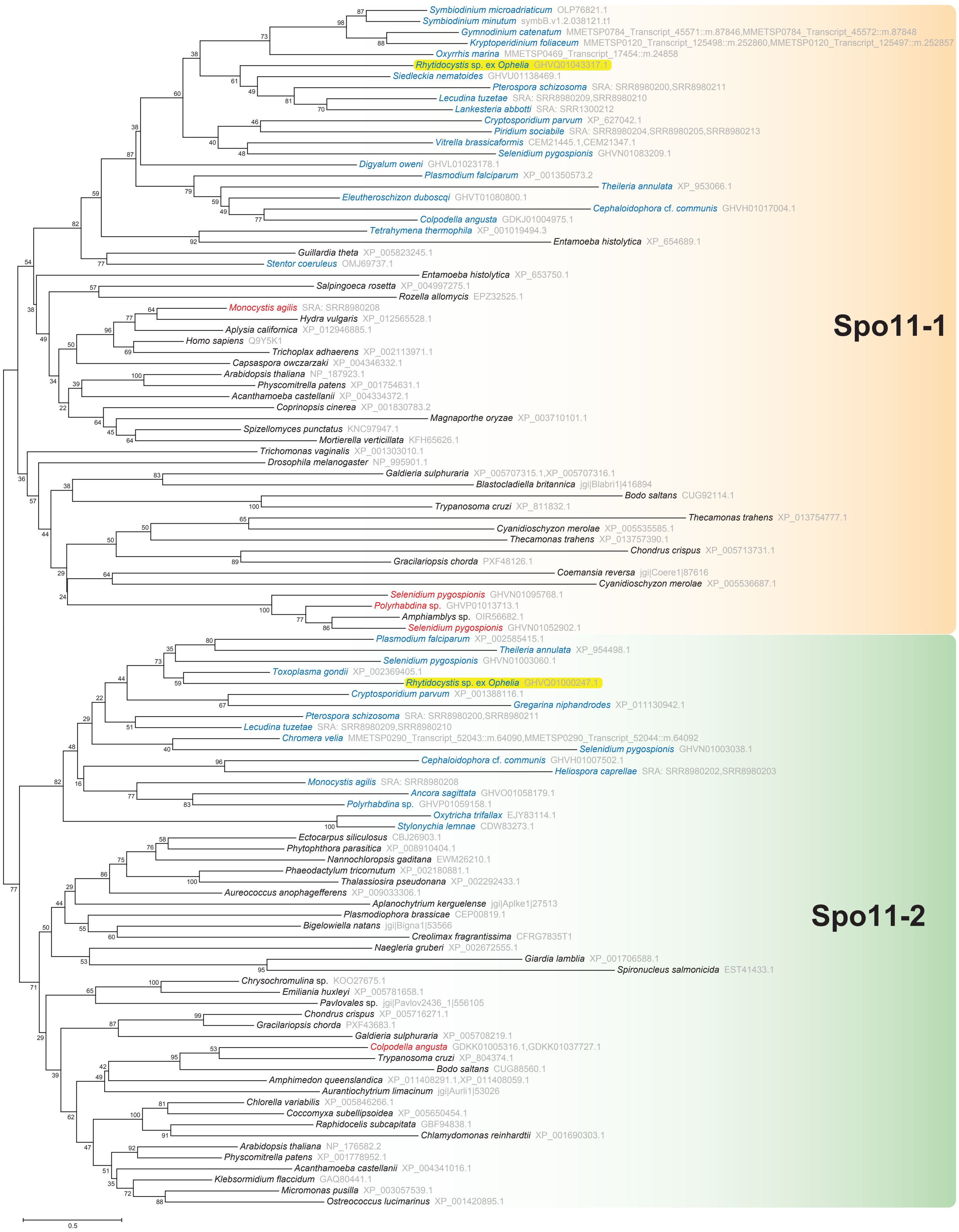

Supplementary Figure S1 (Spo11)

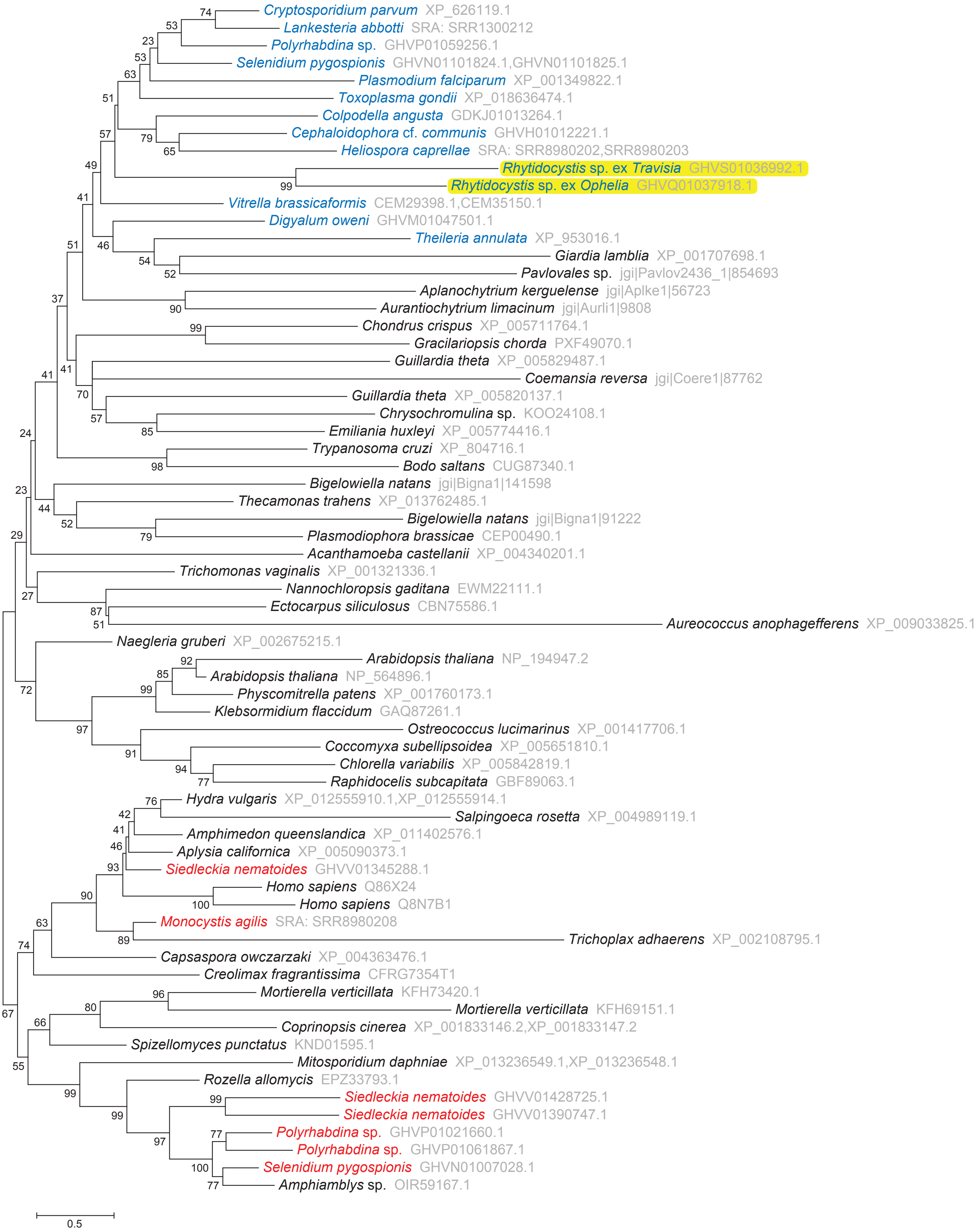

Supplementary Figure S2 (Hop1)

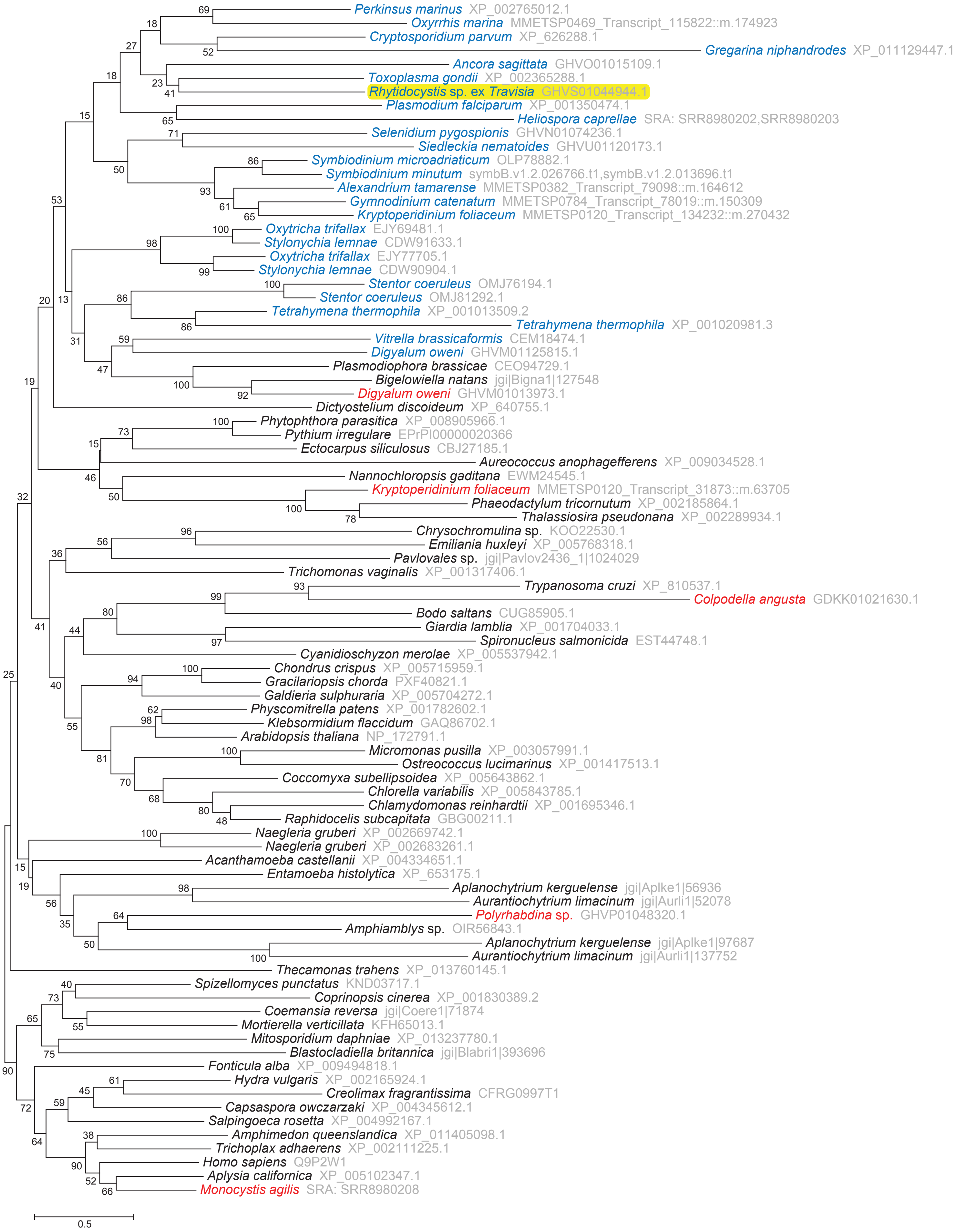

Supplementary Figure S3 (Hop2)

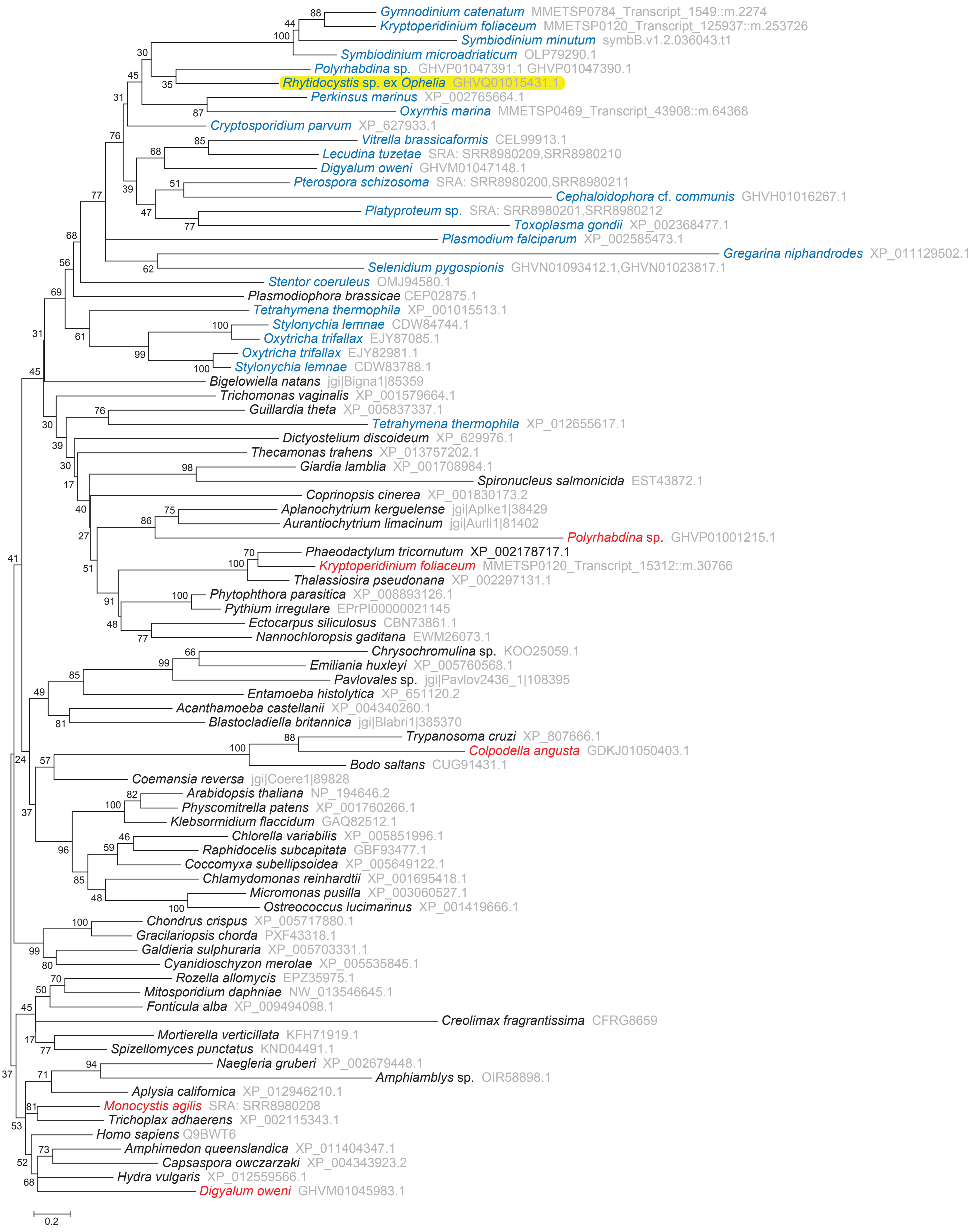

Supplementary Figure S4 (Mnd1)

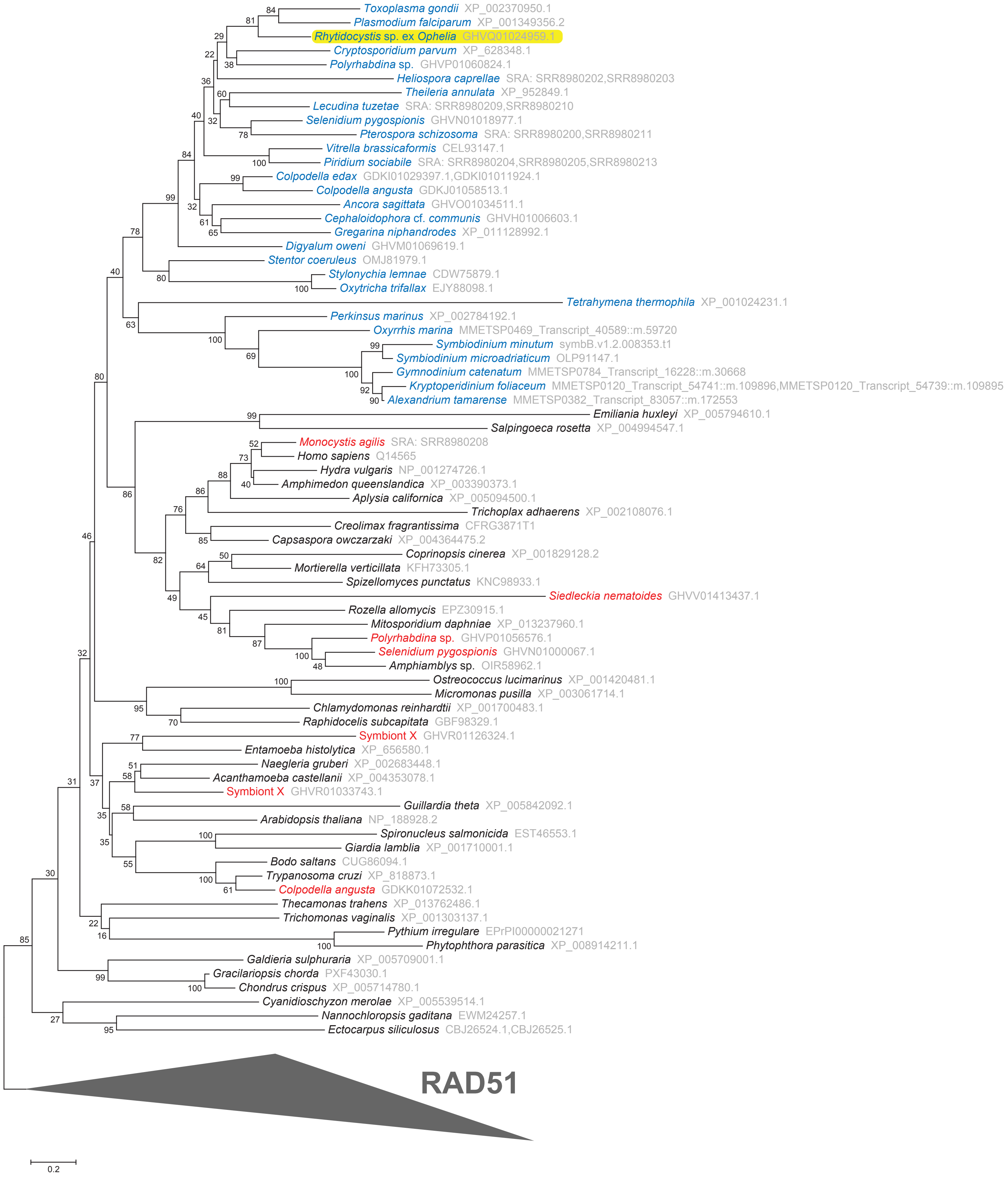

Supplementary Figure S5 (Dmc1)

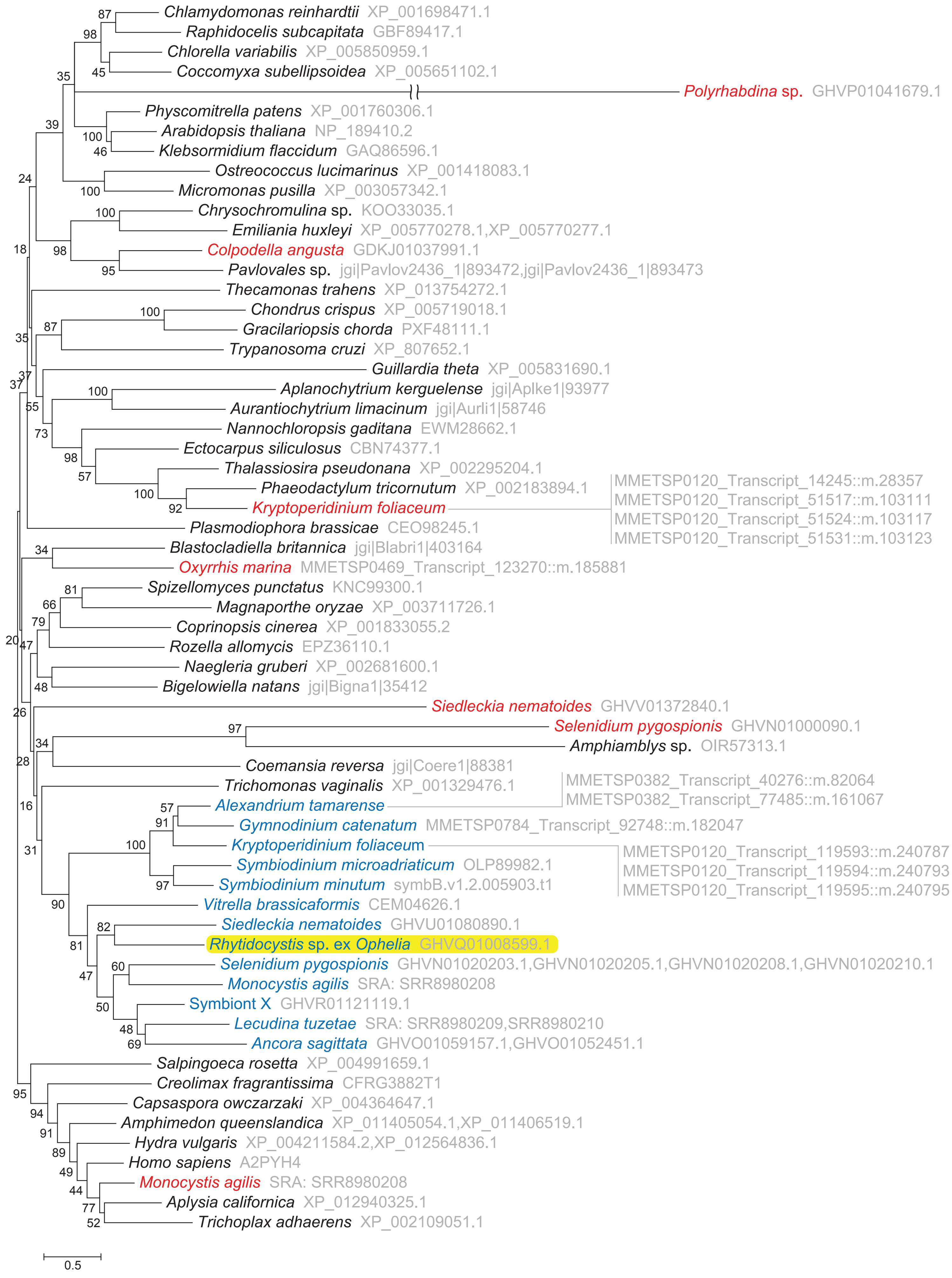

Supplementary Figure S6 (Mer3)

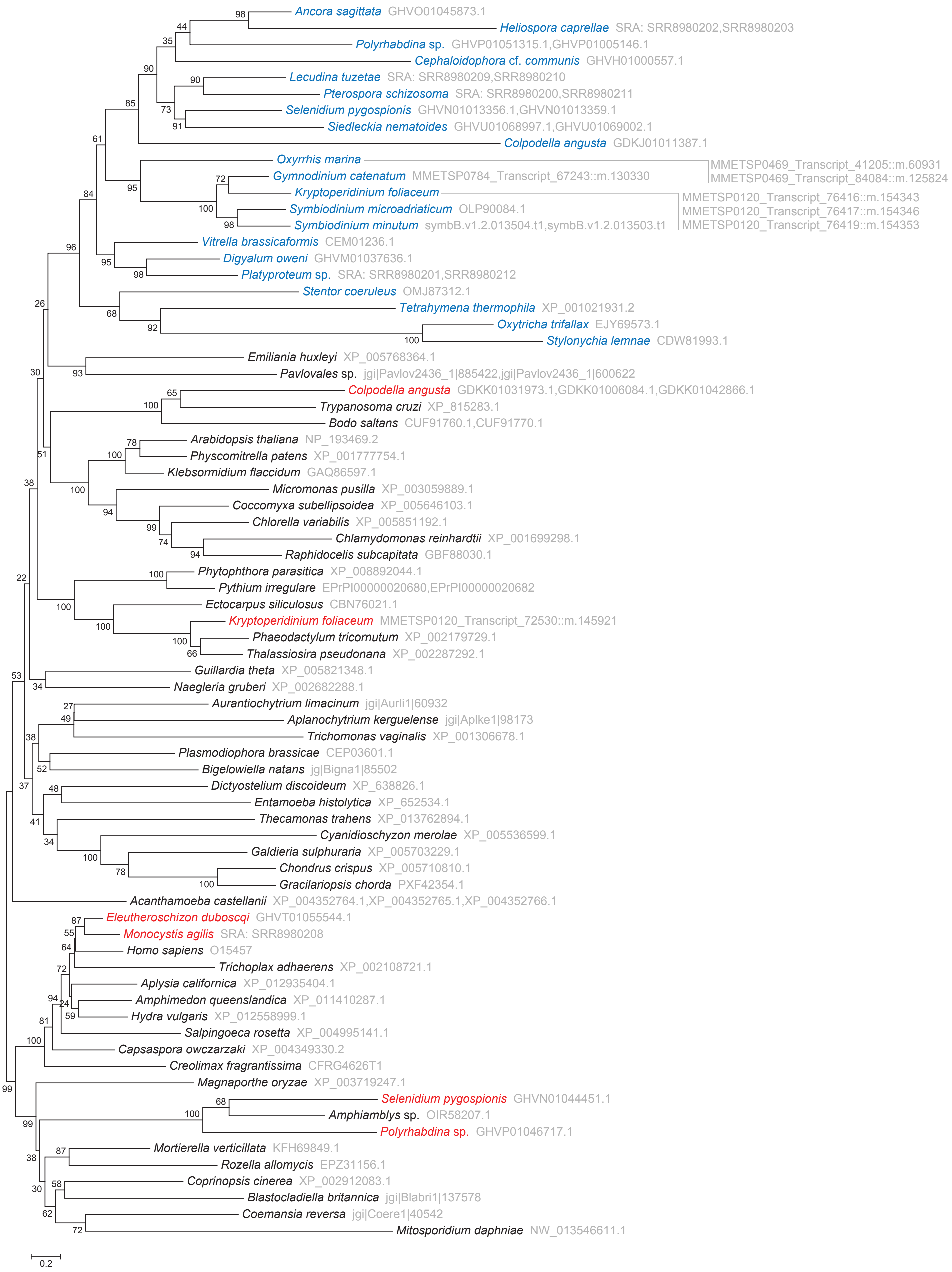

Supplementary Figure S7 (Msh4)

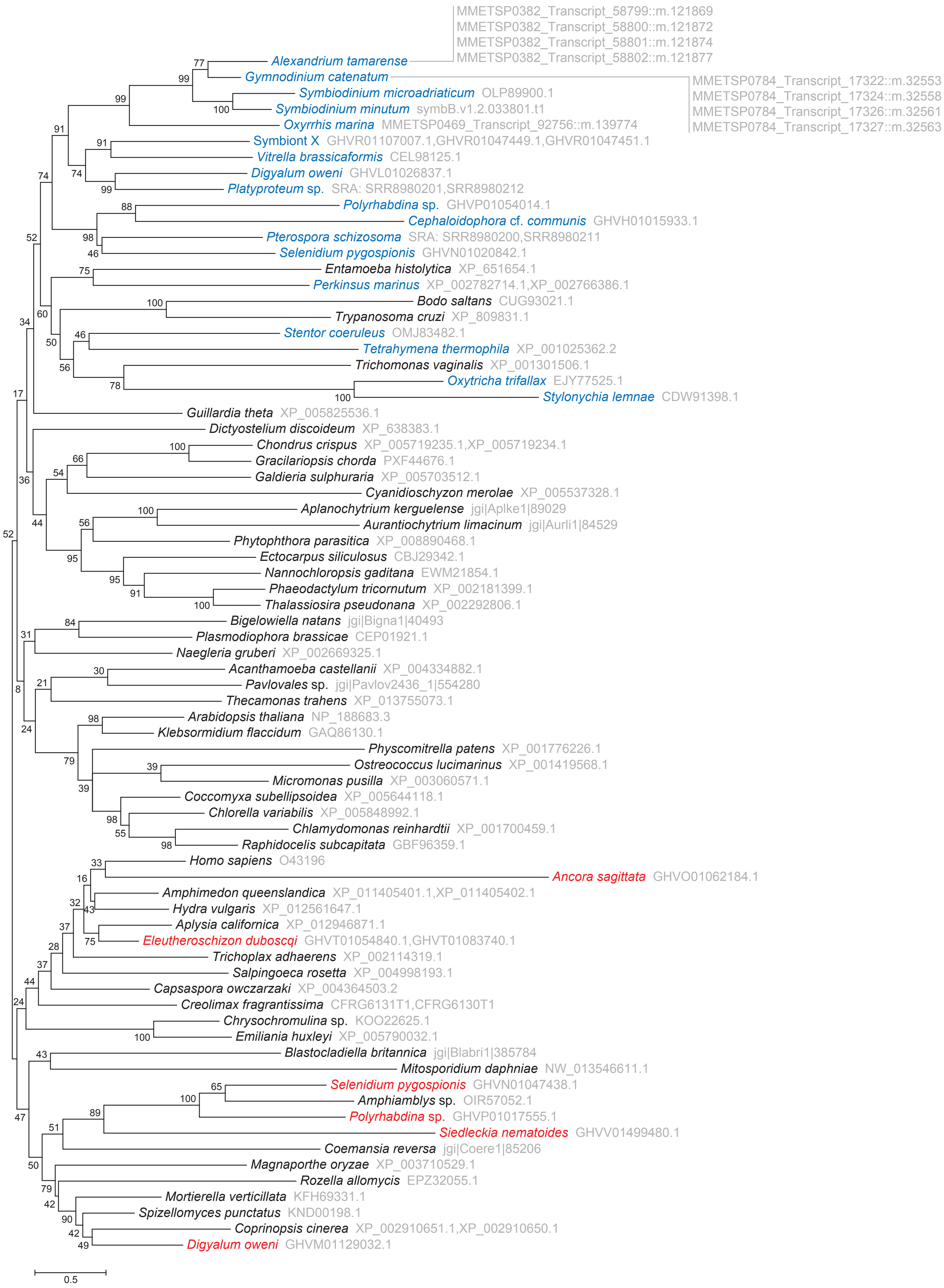

Supplementary Figure S8 (Msh5)

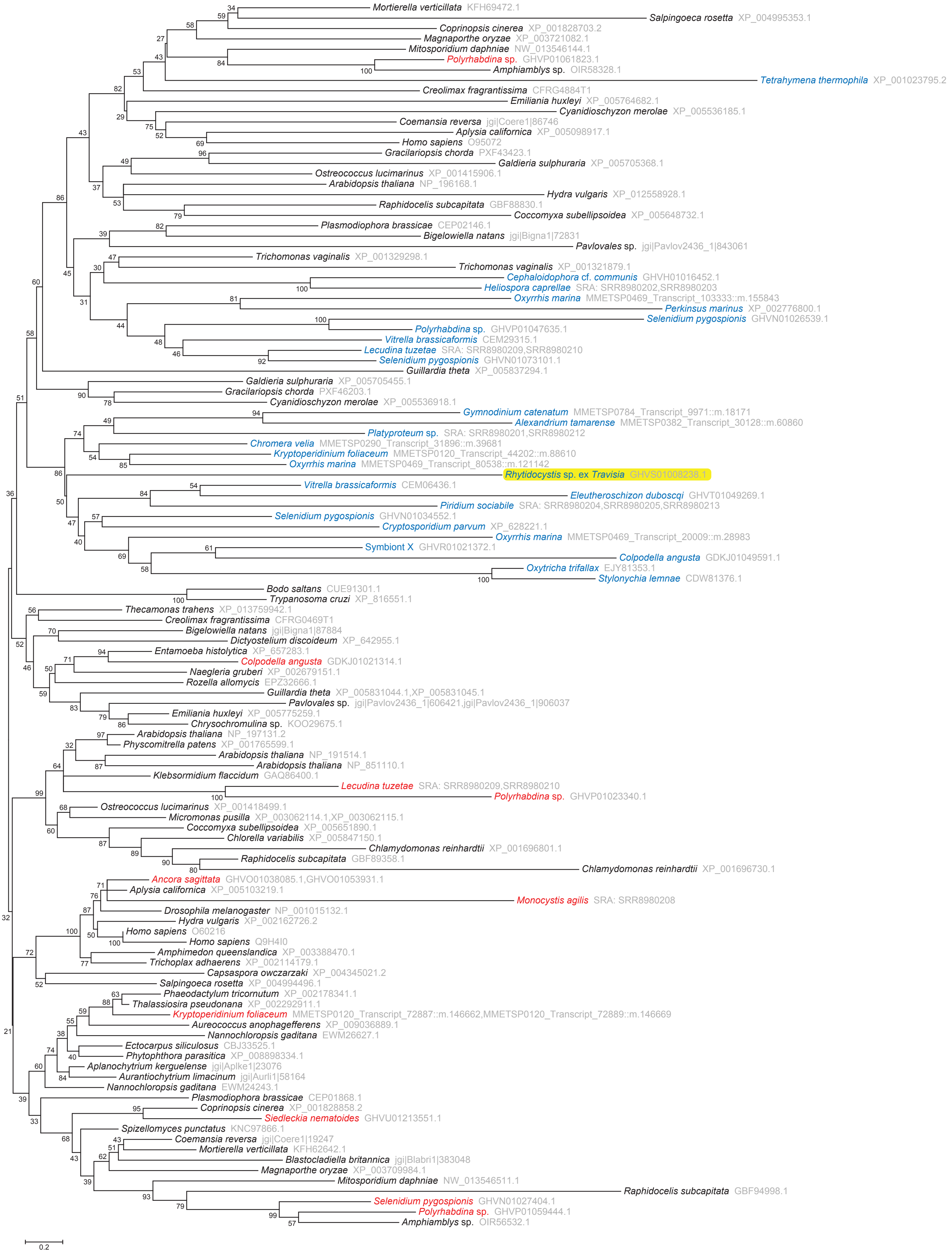

Supplementary Figure S9 (Rad21/Rec8)

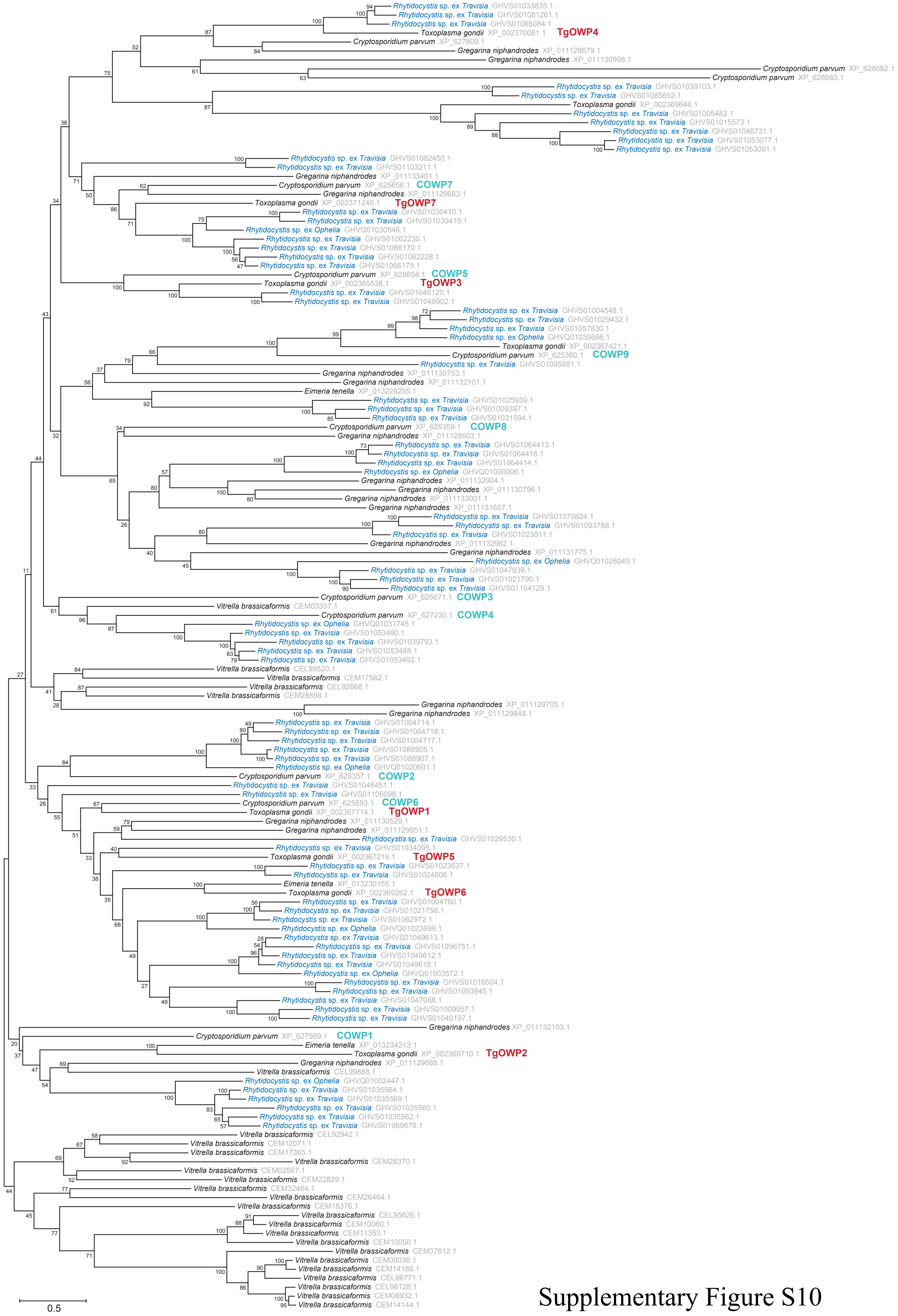

Supplementary Figure S10

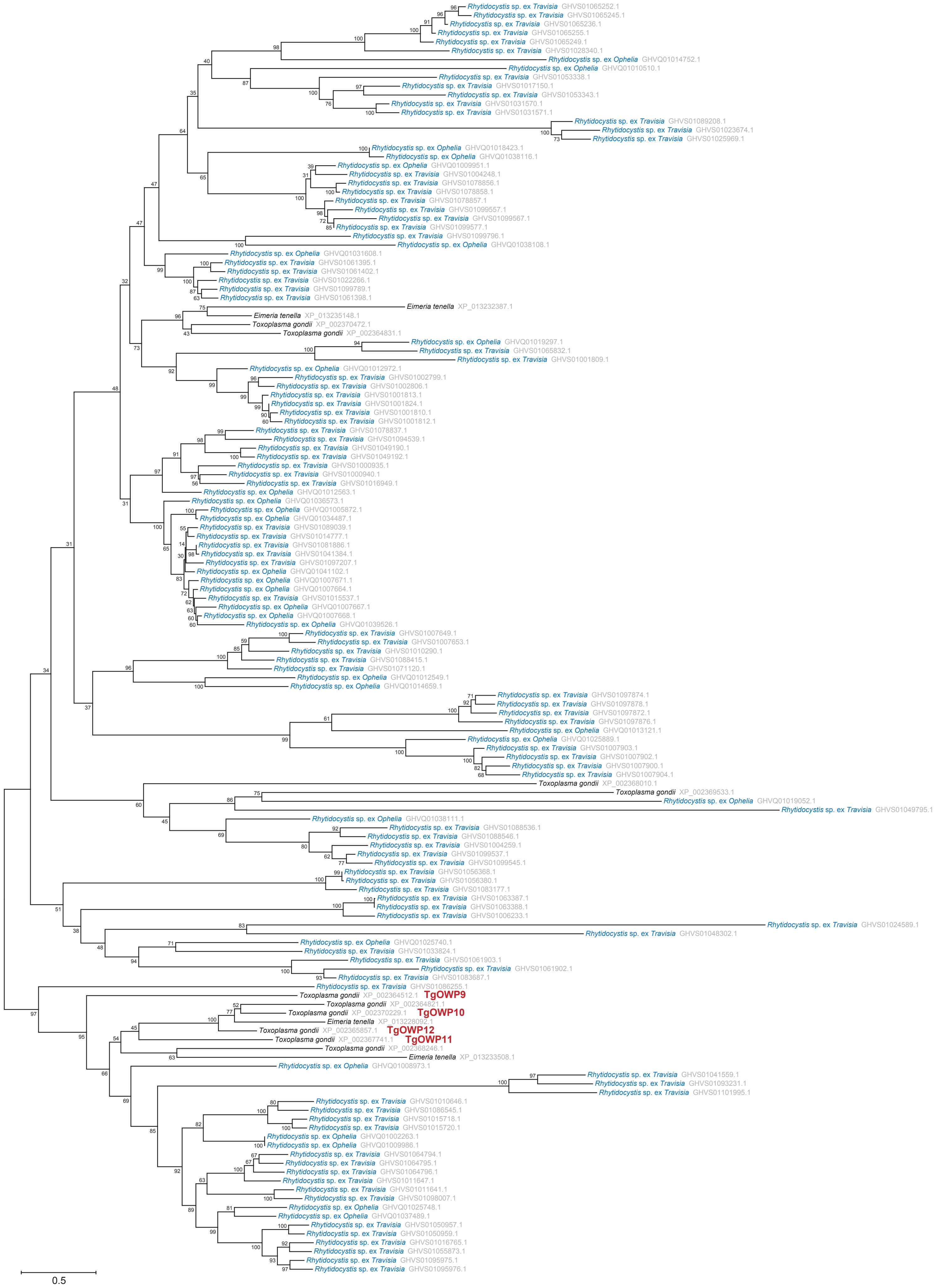

Supplementary Figure S11
